# Development of an In-House Rapid Antimicrobial Susceptibility Testing Protocol for Positive Blood Culture and Its Implementation in Routine Microbiology Laboratories

**DOI:** 10.3389/fmicb.2021.765757

**Published:** 2021-11-30

**Authors:** Min Cao, Lin Huang, Yanyan Hu, Yinfei Fang, Rong Zhang, Gongxiang Chen

**Affiliations:** ^1^Department of Clinical Microbiology, The Second Affiliated Hospital of Zhejiang University School of Medicine, Hangzhou, China; ^2^Department of Clinical Microbiology, Affiliated Jinhua Hospital, Zhejiang University School of Medicine, Jinhua, China

**Keywords:** in-house, rapid antimicrobial susceptibility testing, protocol, bloodstream infection, blood culture, preliminary breakpoint

## Abstract

Bloodstream infections (BSI) are associated with high morbidity and mortality and remain a leading cause of death. Blood culture (BC) including the identification and the antimicrobial susceptibility testing of the causative microorganisms should be performed as soon as possible. In this study, we developed an in-house rapid antimicrobial susceptibility testing (rAST) protocol for positive BC. First, the rAST was performed in the simulated positive BC of standard strains (*Escherichia coli* ATCC 25922, *Staphylococcus aureus* ATCC 25923, and *Pseudomonas aeruginosa* ATCC 27853) at three different times to assess the reproducibility and operability by dispensing four drops of BC broth onto a Mueller–Hinton agar plate after a positive signal. Furthermore, the rAST was performed in clinical positive BCs. The results of rAST at 4, 6, 8, and 18 h of incubation were compared with results of the standard 16- to 20-h disk diffusion method, and the preliminary breakpoints of the rAST method were established according to the inhibition diameter of sensitive strains and resistant strains. Finally, the rAST was performed in the simulated positive BC of clinical strains to evaluate the availability of the preliminary breakpoints. The rAST results of standard strains were distributed evenly at three different times. Among the 202 clinical strains used to establish the preliminary breakpoints, the number of zone diameters that could be read and interpreted (60, 87, 98, and 100%) increased with incubation time (4, 6, 8, and 18 h), and the categorical agreement was acceptable, with total error rates of 3.0, 2.3, 2.1, and 1.3% at 4, 6, 8, and 18 h of incubation, respectively. In conclusion, the in-house rAST protocol for positive BC can be implemented in routine laboratories. It provides reliable antimicrobial susceptibility testing results for BSI pathogens after 4–6 h of incubation.

## Introduction

Bloodstream infections (BSI) are associated with high morbidity and mortality and remain a leading cause of death (Angus et al., 2001). In our hospital, an estimated burden of 6‰ (six every 1,000 inpatients) episodes of BSI happens each year. The early administration of appropriate antibiotics in patients with BSI, which is highly dependent on the identification and susceptibility testing of the pathogens (Altun et al., 2015), improves mortality, reduces the length of hospital stay, and limits the development of resistance (Huang et al., 2013; Sherwin et al., 2017). It was demonstrated decades ago that results of the disk diffusion (DD) method can be read after very short incubation times (Kluge, 1975; Coyle et al., 1984). However, it was not until 2018 that EUCAST established standardized procedures and breakpoints for abbreviated incubation (EUCAST, 2018a,b). The published breakpoints are only authorized for rapid antimicrobial susceptibility testing (rAST) directly from positive blood culture (BC) bottles. However, most laboratories in China follow standards published by the Clinical and Laboratory Standards Institute (CLSI). The CLSI has established rAST directly from positive BC bottles with a standard incubation of 16 to 18 h recently, but it only includes six kinds of antibiotics against Enterobacterales. This paper describes an in-house rAST method directly from positive BC bottles based on CLSI standard DD method but with a shorter incubation time (reading of results after 4, 6, and 8 h), covering a wide range of antibiotics and applicable for more bacteria.

## Materials and Methods

### Standard Procedures for Identification

Microorganisms from positive BC bottles were initially assessed using Gram stain. Species identification was determined by matrix-assisted laser desorption/ionization–time-of-flight mass spectrometry (Bruker Daltonik, Bremen, Germany) using positive BC broth directly (Huang et al., 2019).

### Standard Procedure for Rapid Antimicrobial Susceptibility Testing

#### Media and Disks

Standard CLSI media and Mueller–Hinton agar (MHA) were used. AST was performed on 90-mm circular plates produced in-house using agar from Oxoid (Thermo Fisher Scientific, Basingstoke, United Kingdom). Aerobic (BACT/ALERT^®^ FA, LOT 0001056014) and anaerobic (BACT/ALERT^®^ SN, LOT 0001055094) BC bottles (bioMerieux, Marcy, France). Antibiotic disks (Oxoid, Thermo Fisher Scientific, Basingstoke, United Kingdom) were chosen to represent relevant agents or agent groups used in the treatment of BSI (Table 1).

**TABLE 1:** Antimicrobial agents included and their disk content.

Antimicrobial agents	Disk content	*E. coli* ATCC25922	*P. aeruginosa* ATCC 27853	*S. aureus* ATCC25923
Ampicillin	10 μg	✓		
Cefazolin	30 μg	✓		
Cefotaxime	30 μg	✓		
Aztreonam	30 μg	✓	✓	
Piperacillin-tazobactam	100–10 μg	✓	✓	
Meropenem	10 μg	✓	✓	
Gentamicin	10 μg	✓	✓	✓
Trimethoprim- sulfamethoxazole	1.25–23.75 μg	✓		✓
Levofloxacin	5 μg	✓		
Cefepime	30 μg	✓	✓	
Cefoxitin	30 μg	✓		✓
Cefuroxime	30 μg	✓		
Ceftazidime-avibactam	30–20 μg	✓	✓	
Imipenem	10 μg	✓	✓	
Ciprofloxacin	5 μg		✓	
Erythromycin	15 μg			✓
Clindamycin	2 μg			✓
Linezolid	30 μg			✓

#### Procedure

First, we simulated positive BC with standard strains (*Escherichia coli* ATCC 25922, *Staphylococcus aureus* ATCC 25923, and *Pseudomonas aeruginosa* ATCC 27853). BC bottles were inoculated with 1 ml of one of the culture of the abovementioned species from a solution of 100–200 colony-forming units/ml (suspension adjusted to a turbidity equivalent to 0.5 McFarland standard and then diluted 1:1,000,000) together with 5 ml of blood. According to the performance standards published by the Clinical and Laboratory Standards Institute [CLSI] (2021), the rAST method was performed following a positive signal of the BC bottle by dispensing four drops of BC broth onto an MHA plate. Each standard strain was injected into six BC bottles at a time and repeated three times. The rAST results were read after 4, 6, 8, and 18 h of incubation, respectively. The rAST results of standard strains were used to evaluate the reproducibility and operability of the rAST method. We tested different antibiotics according to the standard strains (Table 1). The species/agent combinations of rAST results were deleted and were not tested for clinical strains when they varied widely in three different times or when less than 50% species/agent combinations could be read in all three times. Meanwhile, the standard quality control was performed to control the media and disks used.

Secondly, we implemented the rAST method from February 1, 2021, to June 1, 2021, for the clinical positive BC samples that were identified by the rapid identification as *Klebsiella pneumoniae*, *E. coli*, *P. aeruginosa*, or *S. aureus*—the reported top four pathogens of BSI (Mayr et al., 2014; Musicha et al., 2017; Hu et al., 2019). At the same time, the positive BC broth was transferred into blood agar plate and incubated overnight. The AST of bacterial colonies incubated overnight was conducted using standard DD method (Clinical and Laboratory Standards Institute [CLSI], 2021). We confirmed the AST result again with standard DD method and broth microdilution method simultaneously when the diameter fell at the breakpoint or in the scope of intermediate. We set the preliminary breakpoints of the rAST method according to the inhibition diameter of sensitive strains and resistant strains.

Finally, we collected clinical strains with different resistance phenotypes, which included *E. coli* (*n* = 13), *K. pneumoniae* (*n* = 22), *S. aureus* (*n* = 25), and *P. aeruginosa* (*n* = 32). We evaluated the preliminary breakpoints by simulating positive BC with these strains by the method described above.

#### Data Analysis

The proportion of readable inhibition zones at each incubation time was calculated for all species/agent combinations. We set the preliminary breakpoints for the rAST method based on data from clinical positive BC strains. A zone diameter is determined as follows: not readable (poor growth), or readable and categorized as susceptible (S), intermediate (I), or resistant (R). For each species, rAST results were interpreted referring to the preliminary breakpoints. The categorical errors were calculated using the standard DD method (Clinical and Laboratory Standards Institute [CLSI], 2021) as the reference, and they were defined as very major error (VME; rAST = S and reference = R), major error (ME; rAST = R and reference = S), or minor error (mE; rAST = S or R and reference = I, rAST = I, and reference = S or R). The acceptable categorical rates of VME, ME, and mE are ≤1.5%, ≤3%, and ≤10%, respectively (Jean et al., 2017). The categorical agreement was defined because the results of rAST were the same as that of reference.

## Results

### Rapid Antimicrobial Susceptibility Testing Results of Standard Strains

The rAST results of standard strains were distributed evenly in three different times (Supplementary Tables 1–3). Among the readable zone of 4 h of incubation, less than 50% of standard *E. coli* ATCC 25922 could be read for four kinds of antibiotics (piperacillin-tazobactam, trimethoprim-sulfamethoxazole, aztreonam, and levofloxacin). Few or no species/agent combinations of standard *S. aureus* ATCC 25923 and *P. aeruginosa* ATCC 27853 could be read except cefoxitin. Among the readable zone of 6 h of incubation, less than 50% of standard *P. aeruginosa* ATCC 27853 could be read for three kinds of antibiotics (meropenem, gentamicin, and imipenem). After 8 h of incubation, all species/agent combinations could be read.

### Establishment of the Preliminary Rapid Antimicrobial Susceptibility Testing Breakpoints

According to the species/agent combinations confirmed above, we tested them in the rAST of 110 clinical positive BCs, which included *E. coli* (*n* = 24), *K. pneumoniae* (*n* = 31), *P. aeruginosa* (*n* = 30), and *S. aureus* (*n* = 25). We classified the positive BC strains as sensitive, intermediate, and resistant strains according to the AST result of bacterial colonies incubated overnight. Among the rAST results, the diameter of resistant strains was lower than that of sensitive strains obviously (Figure 1). For most species/agent combinations, a reliable distinction between sensitive and resistant strains could be achieved (Figure 1). The preliminary breakpoints of the rAST method were established (Supplementary Figures 1–27) according to the rAST results of sensitive strains and resistant strains, and we took it as rapid processing of positive BC in our laboratory (Figure 2).

**FIGURE 1:**
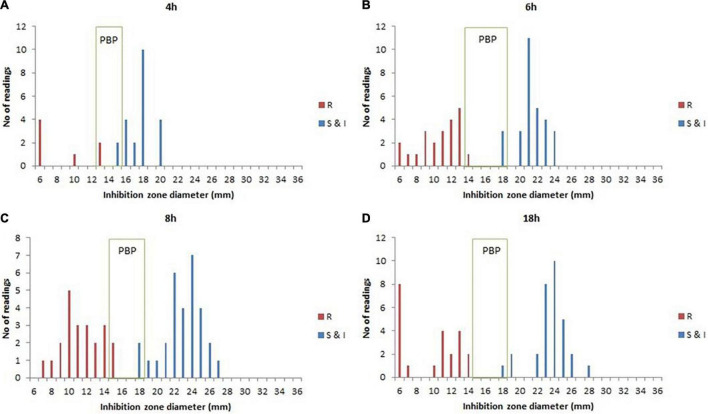
The rapid antimicrobial susceptibility testing (rAST) of *E. coli* and *K. pneumoniae* with imipenem between sensitive strain and resistant strain and the preliminary breakpoint (PBP) distribution. **(A)** 4 h of incubation. **(B)** 6 h of incubation. **(C)** 8 h of incubation. **(D)** 18 h of incubation. R, resistant strains; S&I, sensitive and intermediate strains.

**FIGURE 2:**
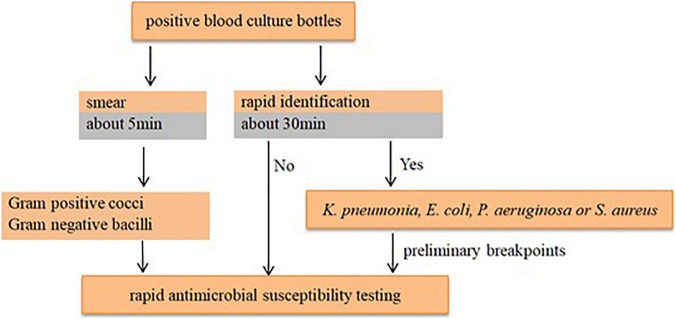
The protocol of rapid processing of positive blood culture in our laboratory.

### Rapid Antimicrobial Susceptibility Testing Results of Clinical Strains

The total number of clinical positive BC bottles included in our experiment was 202. Among them, 92 strains were used to evaluate the preliminary breakpoints set above, which included *E. coli* (*n* = 13), *K. pneumoniae* (*n* = 22), *P. aeruginosa* (*n* = 32), and *S. aureus* (*n* = 25). The number of zone diameters that could be read and interpreted (60, 87, 98, and 100%) increased with incubation time (4, 6, 8, and 18 h; Table 2). Referring to the preliminary breakpoint, the proportion of VME was lower than 1.5% except for linezolid with *S. aureus* (Table 3). No ME was found in the whole study. The proportion of mE was lower than 10% and mainly distributed in cefepime with *E. coli*, *K. pneumoniae*, and gentamicin with *S. aureus* (Table 3). The rAST result of cefoxitin with *S. aureus* could distinguish sensitive strains from resistant strains easily though only about 50% of that could be read at 4 h of incubation. Inducible clindamycin resistance in all *S. aureus* could be detected after 6 h of incubation. In total, the proportion of readable zones increased as incubation time extended.

**TABLE 2:** Theoretical and actual numbers of tests that could be read after 4, 6, 8, and 18 h of incubation and the categorical errors with rAST (in-house preliminary breakpoint).

		Incubation Time (h)			
		4	6	8	18
Theoretical number of tests		897	1817	2003	2003
Readable zones (% of completed tests)		536 (60)	1575 (87)	1961 (98)	2003 (100)
*E. coli* (*n* = 37) and *K. pneumonia* (*n* = 53)					
Theoretical number of tests		847	1207	1207	1207
Readable zones (% of completed tests)		512 (60)	1194 (99)	1207 (100)	1207 (100)
Errors (%)	VME	4 (0.8)	0.0	6 (0.5)	5 (0.4)
	ME	0.0	0.0	0.0	0.0
	mE	12 (2.3)	22 (1.8)	14 (1.2)	11 (0.9)
Categorical agreement (%)		96.9	98.2	98.3	98.7
*P. aeruginosa* (*n* = 62)					
Theoretical number of tests	/	/	310	496	496
Readable zones (% of completed tests)	/	/	142 (46)	469 (95)	496 (100)
Errors (%)	VME	/	0.0	0.0	0.0
	ME	/	0.0	0.0	0.0
	mE	/	3 (2.1)	6 (1.3)	4 (0.8)
Categorical agreement (%)			97.9	98.7	99.2
*S. aureus* (*n* = 50)					
Theoretical number of tests		50	300	300	300
Readable zones (% of completed tests)		24 (48)	239 (80)	285 (95)	300 (100)
Errors (%)	VME	0.0	2 (0.8)	3 (1.1)	3 (1.0)
	ME	0.0	0.0	0.0	0.0
	mE	0.0	9 (3.8)	12 (4.3)	4 (1.3)
Categorical agreement (%)		100	95.4	94.6	97.7

*VME, very major error; ME, major error; and mE, minor error.*

**TABLE 3:** The categorical errors with rAST in clinical evaluating strains.

	4 h				6 h				8 h				18 h			
	VME	ME	mE	*n*	VME	ME	mE	*n*	VME	ME	mE	*n*	VME	ME	mE	*n*
*E. coli* and *K. pneumonia*																
Ampicillin	0	0	2	13	0	0	0	34	0	0	0	35	0	0	0	35
Cefazolin	0	0	0	30	0	0	0	34	0	0	0	35	0	0	0	35
Cefotaxime	0	0	0	30	0	0	0	34	0	0	0	35	0	0	0	35
Aztreonam		NA			0	0	0	34	0	0	1	35	0	0	0	35
Piperacillin-tazobactam		NA			0	0	4	34	0	0	0	35	0	0	0	35
Meropenem	0	0	3	30	0	0	0	34	0	0	0	35	0	0	0	35
Gentamicin	0	0	0	31	0	0	0	34	0	0	0	35	0	0	0	35
Trimethoprim- sulfamethoxazole		NA			0	0	0	34	0	0	0	35	0	0	0	35
Levofloxacin		NA			0	0	0	34	0	0	0	35	0	0	0	35
Cefepime	0	0	2	31	0	0	7	34	0	0	3	35	0	0	0	35
Cefoxitin	0	0	0	31	0	0	3	34	4	0	0	35	4	0	0	35
Cefuroxime	0	0	0	30	0	0	0	34	0	0	0	35	0	0	0	35
Ceftazidime-avibactam	3	0	3	30	0	0	3	34	0	0	4	35	0	0	3	35
Imipenem	0	0	2	31	0	0	0	34	0	0	0	35	0	0	0	35
Total errors (%)	3 (1.0)	0	12 (2.6)	/	0	0	17 (3.6)	/	4 (0.9)	0	8 (1.7)	/	4 (0.9)	0	4 (0.6)	/
*P. aeruginosa*																
Aztreonam		NA			0	0	0	16	0	0	0	30	0	0	0	32
Piperacillin-tazobactam		NA			0	0	2	15	0	0	5	30	0	0	1	32
Meropenem		NA				NA			0	0	0	30	0	0	0	32
Gentamicin		NA				NA			0	0	0	30	0	0	0	32
Cefepime		NA			0	0	0	15	0	0	0	30	0	0	0	32
Ciprofloxacin		NA			0	0	1	16	0	0	1	30	0	0	1	32
Ceftazidime-avibactam		NA			0	0	0	15	0	0	0	30	0	0	0	32
Imipenem		NA				NA						30	0	0	1	32
Total errors (%)		NA			0	0	3 (3.9)		0	0	6 (2.5)		0	0	3 (1.2)	
*S. aureus*																
Cefoxitin	0	0	0	12	0	0	0	21	0	0	0	23	0	0	0	25
Trimethoprim- sulfamethoxazole		NA			0	0	0	21	0	0	0	23	0	0	0	25
Gentamicin		NA			0	0	7	21	0	0	8	23	0	0	0	25
Erythromycin		NA			0	0	0	21	0	0	0	23	0	0	0	25
Clindamycin		NA			0	0	2	21	0	0	2	23	0	0	0	25
Linezolid		NA			2	0	0	20	3	0	0	23	3	0	0	25
Total errors (%)	0	0	0		2 (1.6)	0	9 (7.5)		3 (2.2)	0	10 (7.2)		3 (2.0)	0	0	

*VME, very major error; ME, major error; mE, minor error; and NA, not applicable.*

### The Categorical Errors of Rapid Antimicrobial Susceptibility Testing (18 h) Between the Clinical and Laboratory Standards Institute Standard and In-House Standard

Applying the CLSI breakpoint, the total errors of the rAST results of clinical *E. coli* and *K. pneumoniae* strains were higher, but no VME was found (Table 4). At the same time, the breakpoint was too loose for sensitive strains. In most sensitive strains of our experiment, the inhibition zone diameter of the rAST (18 h of incubation) was lower than that of the reference method (standard DD method with bacterial colonies incubated overnight). It could reduce the total errors referring to our in-house preliminary breakpoint, but one VME appeared in cefotaxime.

**TABLE 4:** The categorical errors with rAST (18 h) between CLSI and in-house standard.

		Ampicillin (*n* = 37)	Aztreonam (*n* = 90)	Cefotaxime (*n* = 90)	Trimethoprim-sulfamethoxazole (*n* = 90)
CLSI M100	ED31				
Breakpoint		13–17	17–21	22–26	10–16
Errors (%)	VME	0.0	0.0	0.0	0.0
	ME	0.0	2 (2.2)	7 (7.8)	0.0
	mE	2 (8.3)	11 (12.2)	3 (3.3)	5 (5.6)
	Total	8.3	14.4	11.1	5.6
In-house					
Breakpoint		12–16	13–16	16–19	10–15
Errors (%)	VME	0.0	0.0	1 (1.1)	0.0
	ME	0.0	0.0	0.0	0.0
	mE	0.0	1 (1.1)	0.0	2 (2.2)
	Total	0.0	1.1	1.1	2.2

*VME, very major error; ME, major error; and mE, minor error.*

## Discussion

With the development of antibiotics resistance, here, in China, the National Health Commission has introduced strict policies to limit the use of antibiotics (He et al., 2019). However, as a life-threatening and multiple organ involvement infections, BSI needs early administration of appropriate antibiotics. Therefore, as a clinical laboratory, identification and AST of the causative microorganisms by BC should be performed as soon as possible (Sherwin et al., 2017).

Recently, the CLSI had published that positive BC broth can be used as the inoculum for direct DD testing of select antibiotics against Enterobacterales with a standard incubation of 16 to 18 h, using current DD breakpoints. Therefore, we evaluated whether this direct DD method could be applied in our laboratory. At the same time, we evaluated more antibiotics for the top four pathogens of BSI in our hospital (*K. pneumoniae*, *E. coli*, *P. aeruginosa*, and *S. aureus*) and for shorter direct incubation time, e.g., 4 and 6–8 h. First, we evaluated the reproducibility and operability of rAST at 4 and 6–8 h with standard strains. We found that standard strain *E. coli* ATCC 25922 was better than standard strains *S. aureus* ATCC 25923 and *P. aeruginosa* ATCC 27853 in the readability of results at 4 h. The inhibition zone diameters of standard strains were all in the quality control ranges. The inhibition zone diameter of rAST was small at 4 h, and became bigger over time but was still smaller than that of original standard strains at 18 h. The reproducibility of the rAST method was acceptable, and we did not find a big deviation in three different times.

We implemented the rAST method for positive BC of *K. pneumoniae*, *E. coli*, *P. aeruginosa*, or *S. aureus* for 4 months. We found that only 60% of *E. coli* and *K. pneumoniae* (4 h of incubation) and 46% of *P. aeruginosa* (6 h of incubation) were readable. Our finding showed that it is necessary to extend the incubation time when the rAST result could not be read at 4 or 6 h, which was also reported by other authors (Chandrasekaran et al., 2018; Périllaud et al., 2019). However, in an instance where a result could not be read after 8 h of incubation, an overnight incubation was needed. A thin growth in the inhibition zone could appear especially at 4 h of incubation. The result could not be read in this situation, and further extension of the incubation time is necessary. This phenomenon may attribute to that the DD and the growth of bacteria did not reach a balance. However, it needed to be ignored when this happened in trimethoprim-sulfamethoxazole according to our findings (Supplementary Figure 28).

We found that the rAST result of clinical strains could easily distinguish sensitive strains from resistant strains. The rAST result of cefoxitin with *S. aureus* could distinguish methicillin resistant *S. aureus* from methicillin-sensitive *S. aureus* easily, which is very important to the clinician. The inhibition zone diameter of rAST at 18 h was smaller than that of bacterial colonies incubated overnight, and more errors appeared using current DD breakpoints in CLSI M100ED31 Table 2A (Clinical and Laboratory Standards Institute [CLSI], 2021). Therefore, the zone diameter breakpoints for the rAST method need to be recalibrated (Fröding et al., 2017; Hombach et al., 2017; van den Bijllaardt et al., 2017). To reduce the operation errors, we set the preliminary breakpoint as wide as possible. The zone diameter of the preliminary breakpoint was lower than that of current DD breakpoints in CLSI M100, which was the same as EUCAST. The total errors were very low and were acceptable (Jean et al., 2017), referring to the preliminary breakpoint established by our laboratory and mainly distributed in cefepime with *E. coli*, *K. pneumoniae*, and gentamicin with *S. aureus*, so the rAST method of these two species/agent combinations needs to be reassessed. We found several VME in the rAST of linezolid resistant *S. aureus*, which might attributed to the plasmid instability. We suggest that the rAST result should not be reported when it fell on the breakpoint or in the intermediate ranges.

## Conclusion

The method for rAST directly from positive BC bottles offers results within a standard workday, and it is not complicated to introduce into standard clinical microbiology laboratories. The rAST method could even be performed according to the smear result if the laboratory is unable to conduct the quick identification. It is cost effective, is more flexible compared with any other system, and is easier to find heterogeneous resistant strains. On the basis of the results of this study, a preliminary set of breakpoints was defined. However, further studies are required to evaluate more strains using different BC bottles, disks, and media to determine the final breakpoints.

## Data Availability Statement

The original contributions presented in the study are included in the article/Supplementary Material, further inquiries can be directed to the corresponding author/s.

## Ethics Statement

All the specimens were anonymized, and patients were not physically involved in this study. No consent was needed for this study.

## Author Contributions

GC conceived and designed the study. MC performed the experiments and wrote the manuscript. LH and YF analyzed the data. LH and YH provided experimental technical support. GC and RZ improved the manuscript. All authors contributed to the article and approved the submitted version.

## Conflict of Interest

The authors declare that the research was conducted in the absence of any commercial or financial relationships that could be construed as a potential conflict of interest.

## Publisher’s Note

All claims expressed in this article are solely those of the authors and do not necessarily represent those of their affiliated organizations, or those of the publisher, the editors and the reviewers. Any product that may be evaluated in this article, or claim that may be made by its manufacturer, is not guaranteed or endorsed by the publisher.
