# Supplementary material for: Development of an In-House Rapid Antimicrobial Susceptibility Testing Protocol for Positive Blood Culture and Its Implementation in Routine Microbiology Laboratories
Source: Front Microbiol. 2021 Nov 30;12:765757. doi: 10.3389/fmicb.2021.765757 (PMC8669140; doi:10.3389/fmicb.2021.765757)
Supplement: Supplementary file 1 [file Presentation_1.PPTX]

## Slide 1
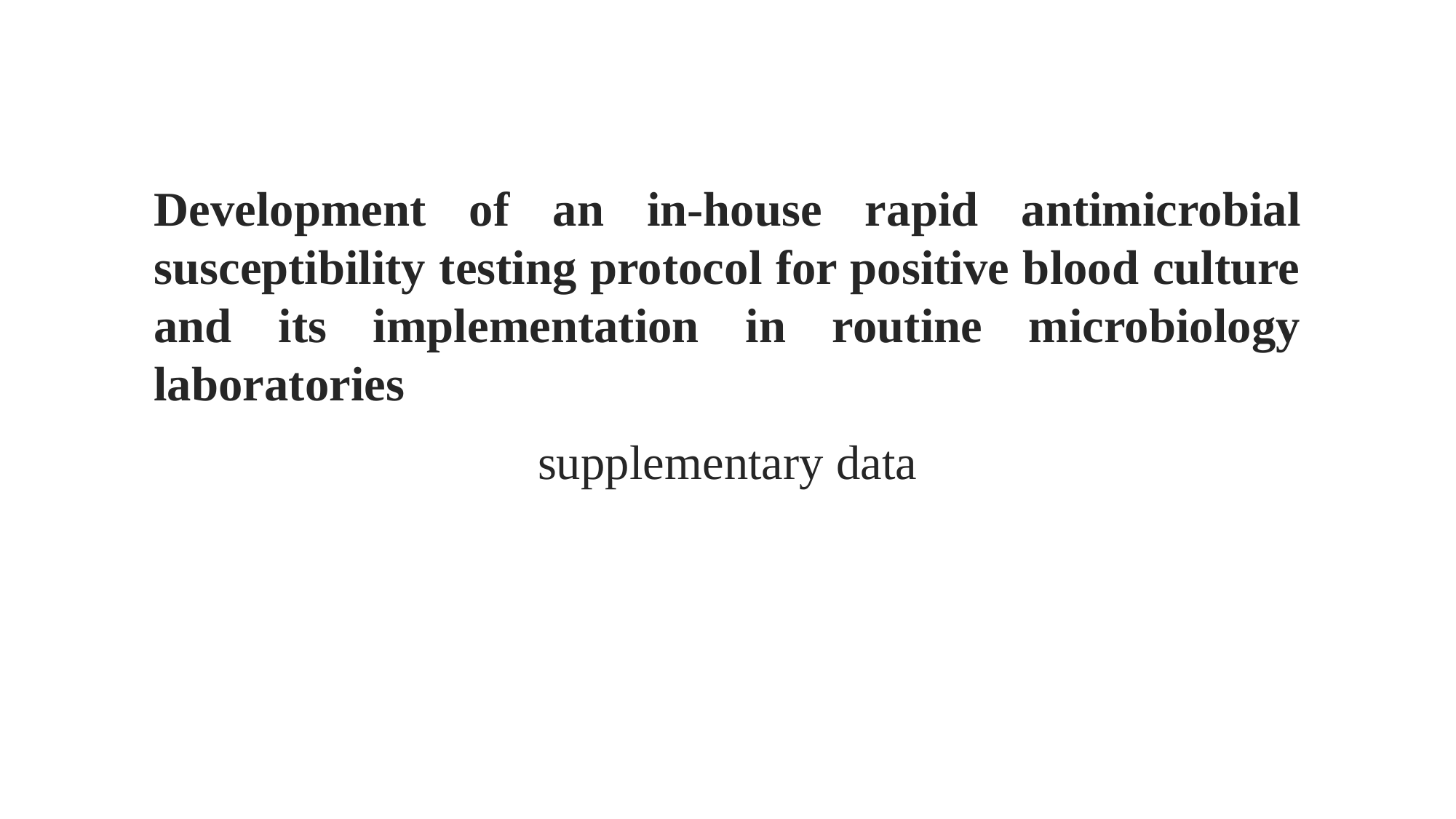

# Development of an in-house rapid antimicrobial susceptibility testing protocol for positive blood culture and its implementation in routine microbiology laboratories
supplementary data

## Slide 2
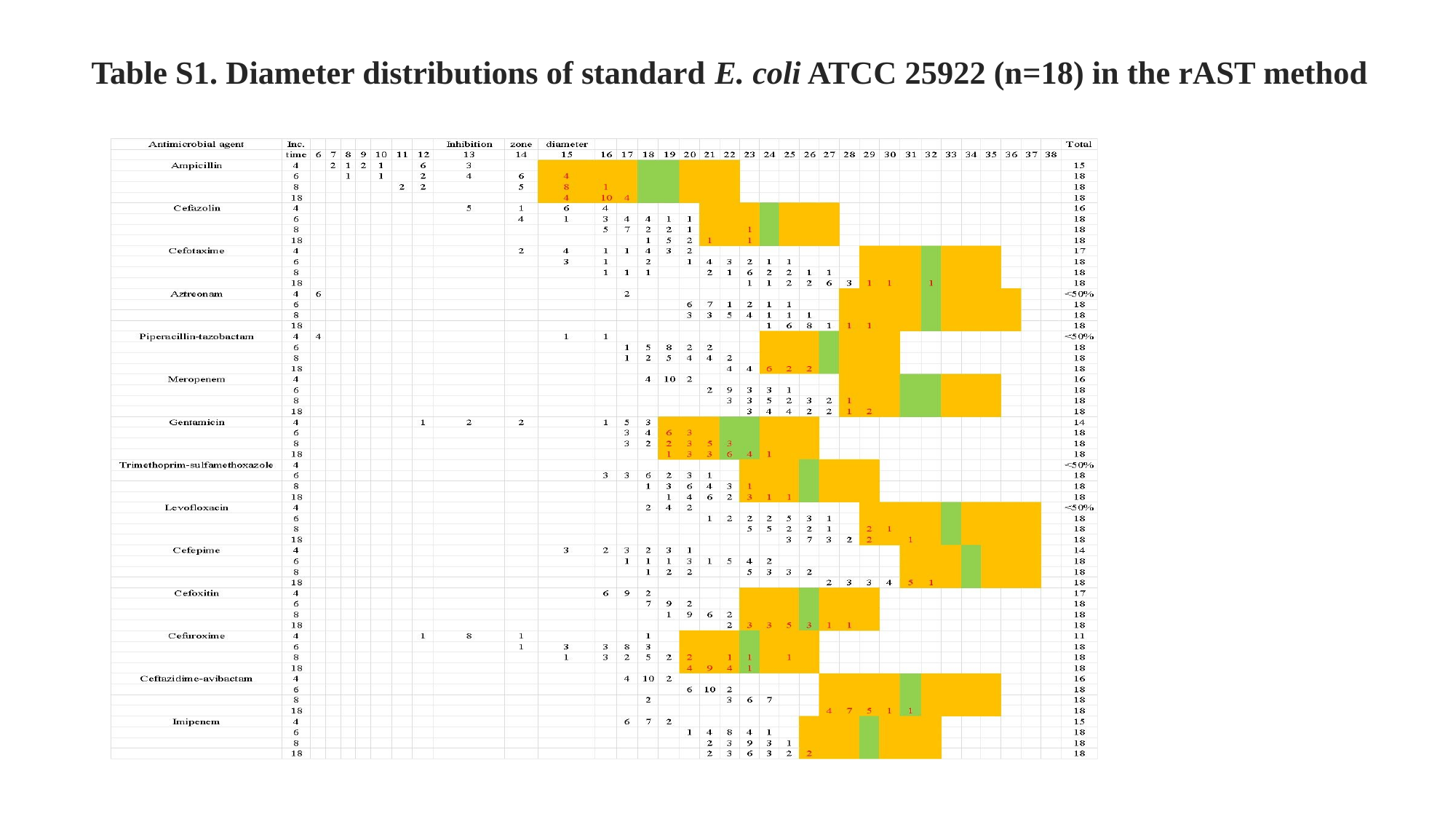

# Table S1. Diameter distributions of standard E. coli ATCC 25922 (n=18) in the rAST method

## Slide 3
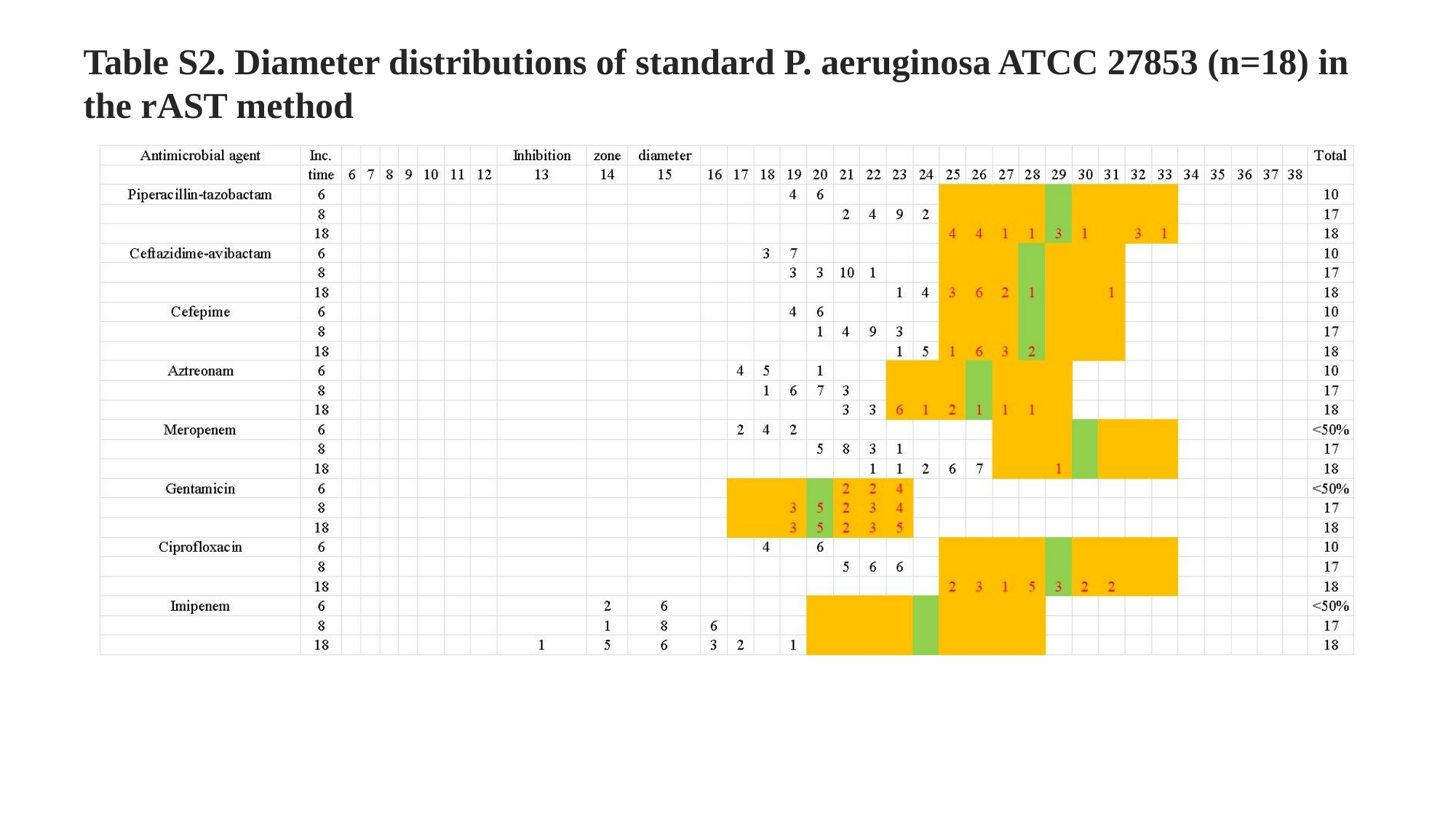

# Table S2. Diameter distributions of standard P. aeruginosa ATCC 27853 (n=18) in the rAST method

## Slide 4
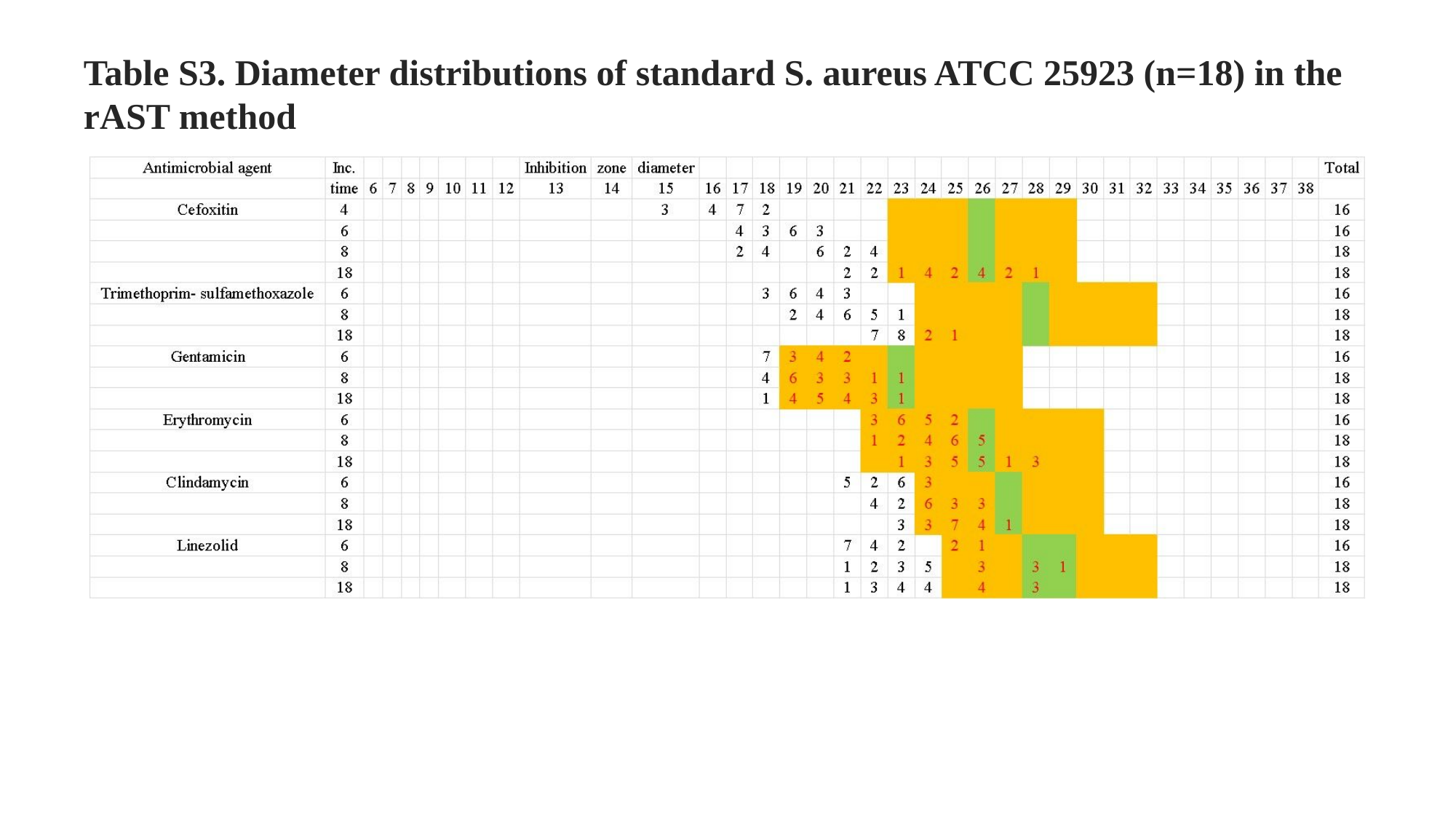

# Table S3. Diameter distributions of standard S. aureus ATCC 25923 (n=18) in the rAST method

## Slide 5
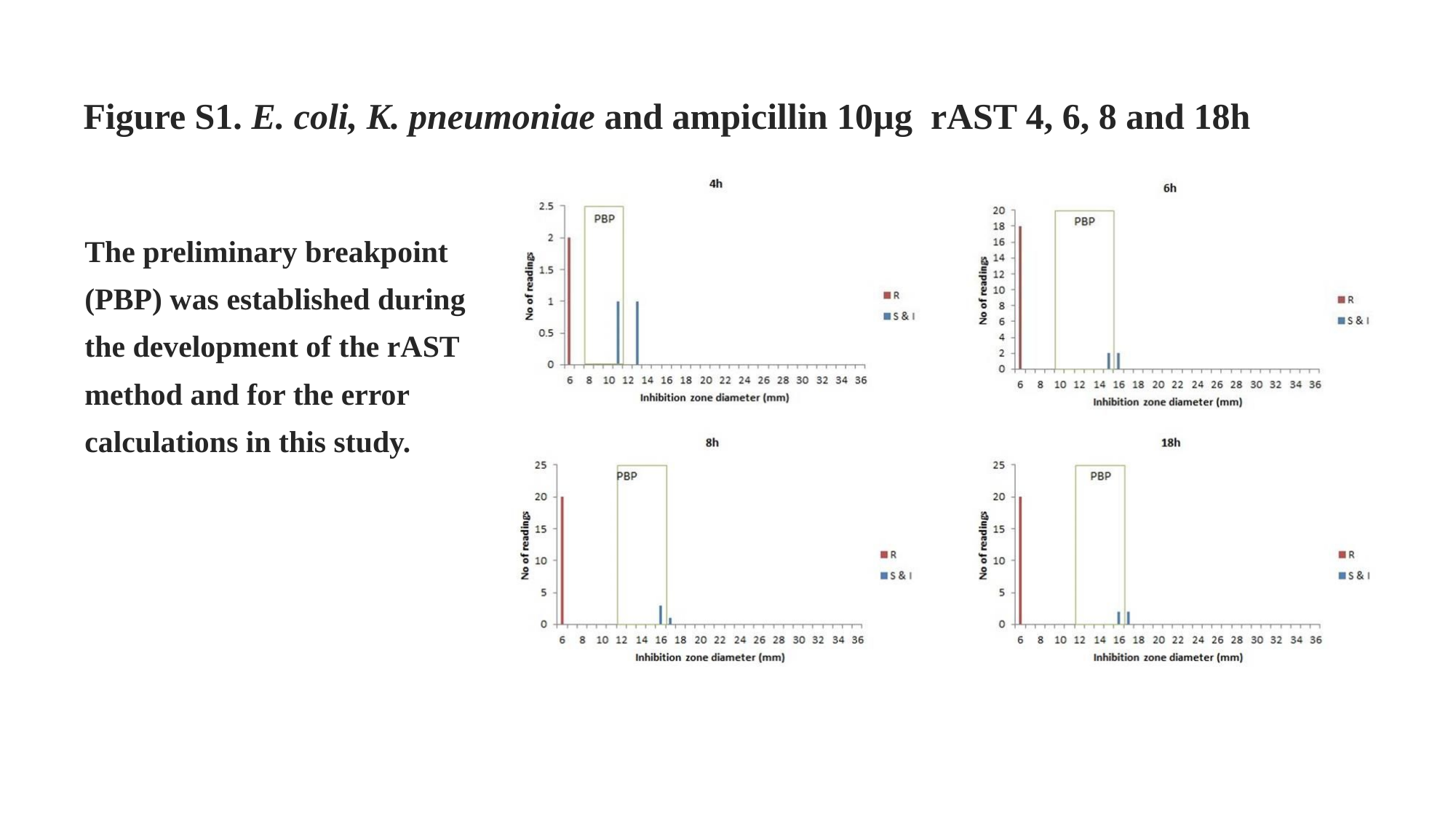

# Figure S1. E. coli, K. pneumoniae and ampicillin 10µg rAST 4, 6, 8 and 18h
The preliminary breakpoint (PBP) was established during the development of the rAST method and for the error calculations in this study.

## Slide 6
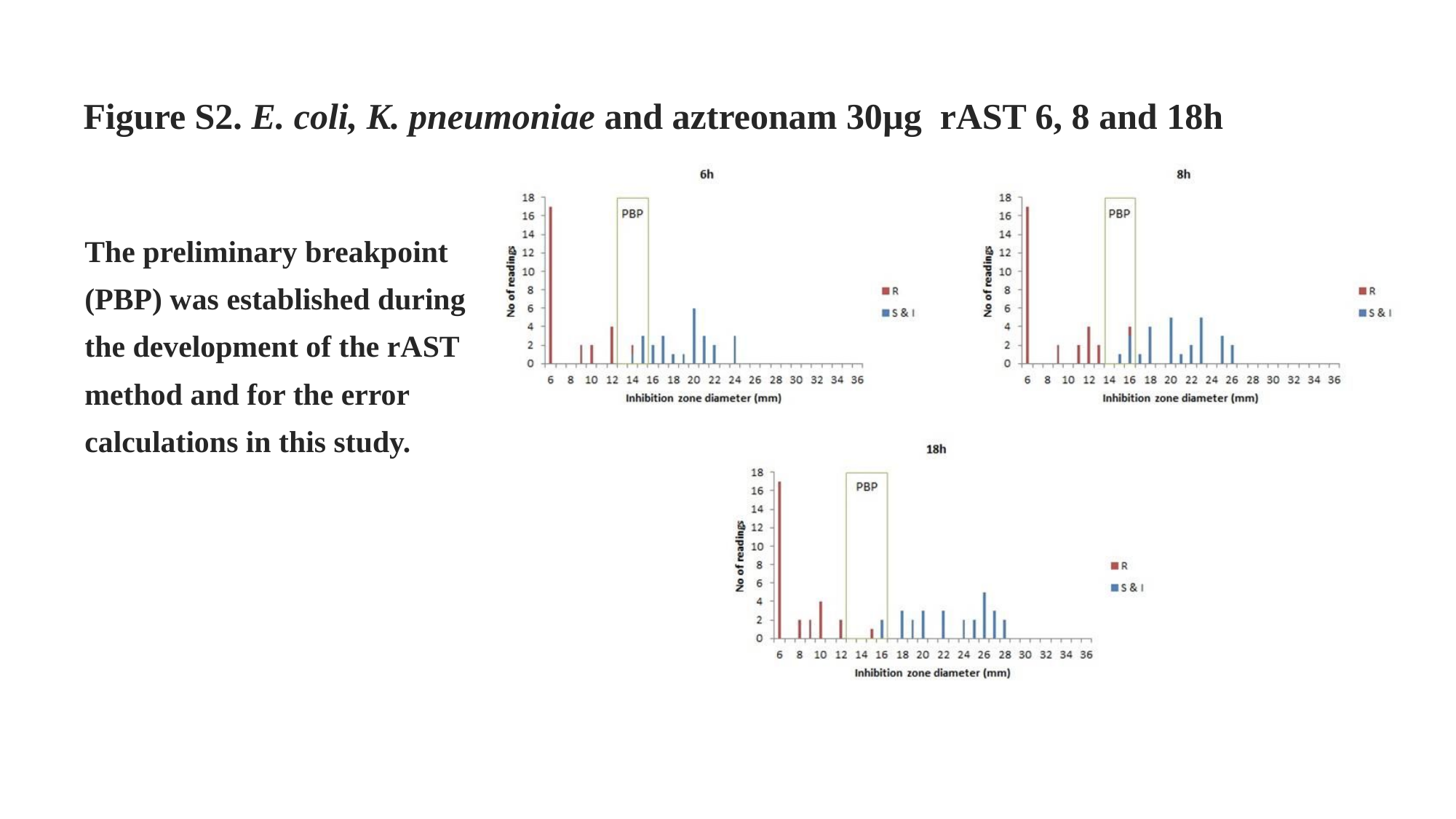

# Figure S2. E. coli, K. pneumoniae and aztreonam 30µg rAST 6, 8 and 18h
The preliminary breakpoint (PBP) was established during the development of the rAST method and for the error calculations in this study.

## Slide 7
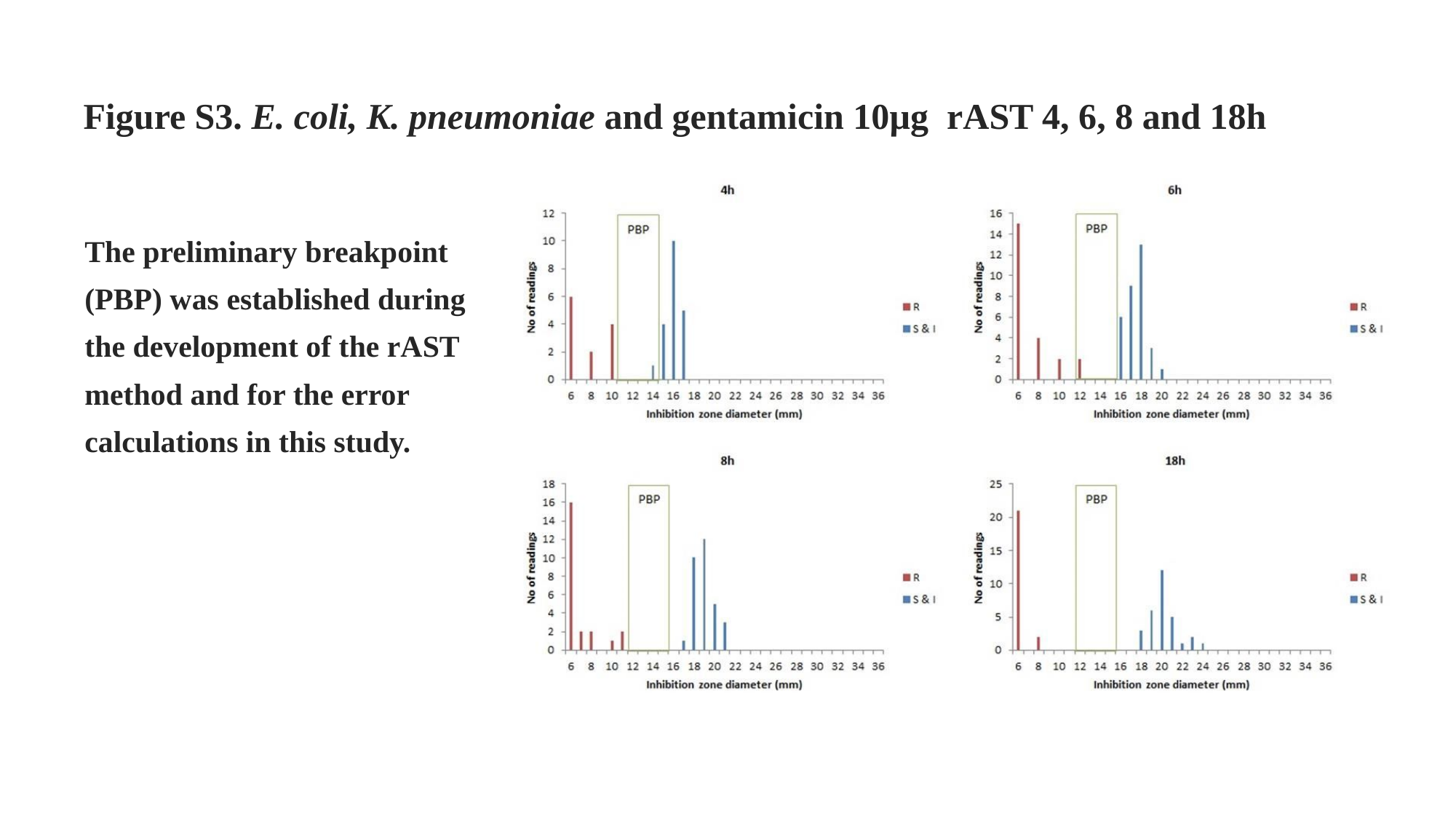

# Figure S3. E. coli, K. pneumoniae and gentamicin 10µg rAST 4, 6, 8 and 18h
The preliminary breakpoint (PBP) was established during the development of the rAST method and for the error calculations in this study.

## Slide 8
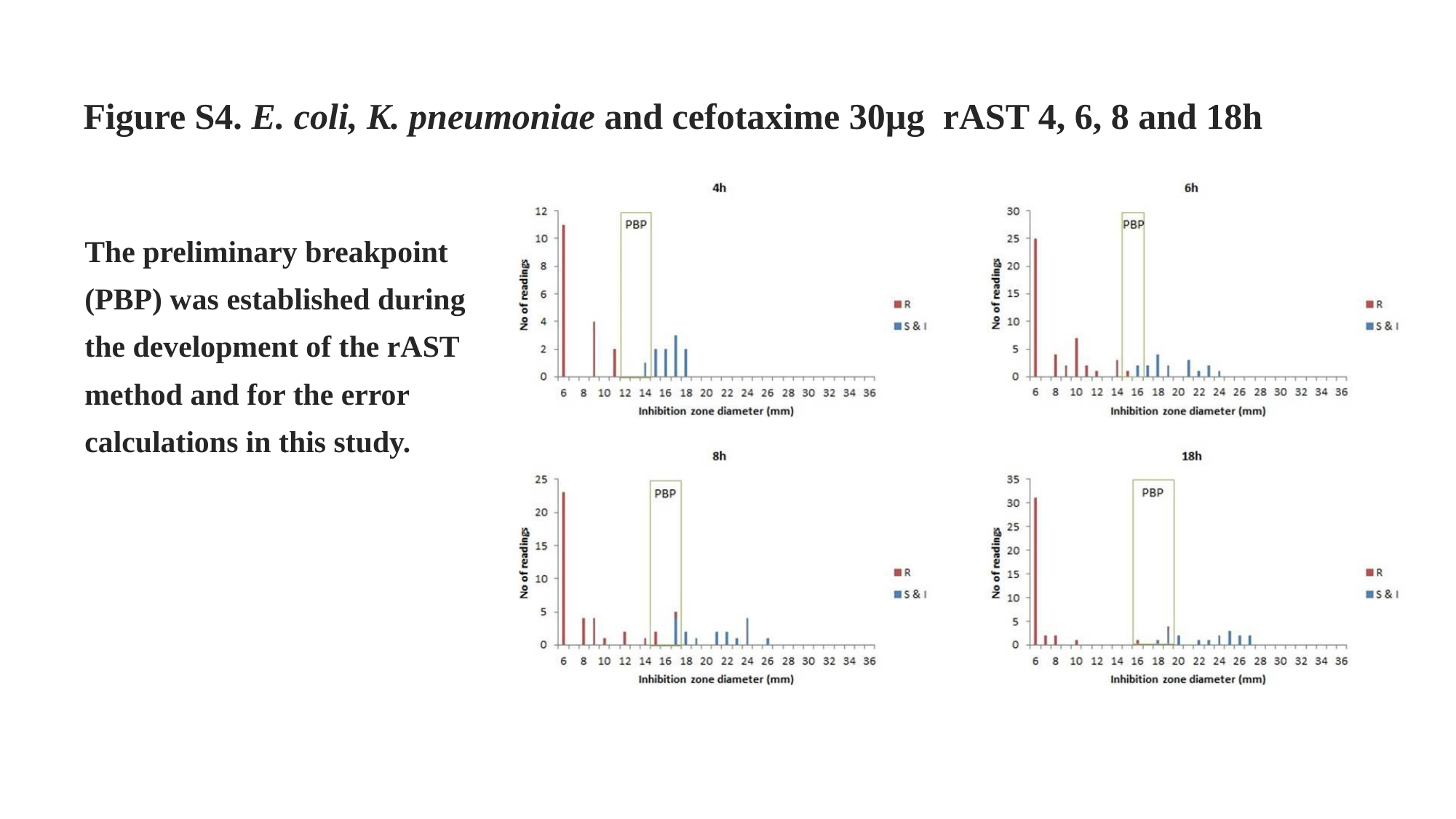

# Figure S4. E. coli, K. pneumoniae and cefotaxime 30µg rAST 4, 6, 8 and 18h
The preliminary breakpoint (PBP) was established during the development of the rAST method and for the error calculations in this study.

## Slide 9
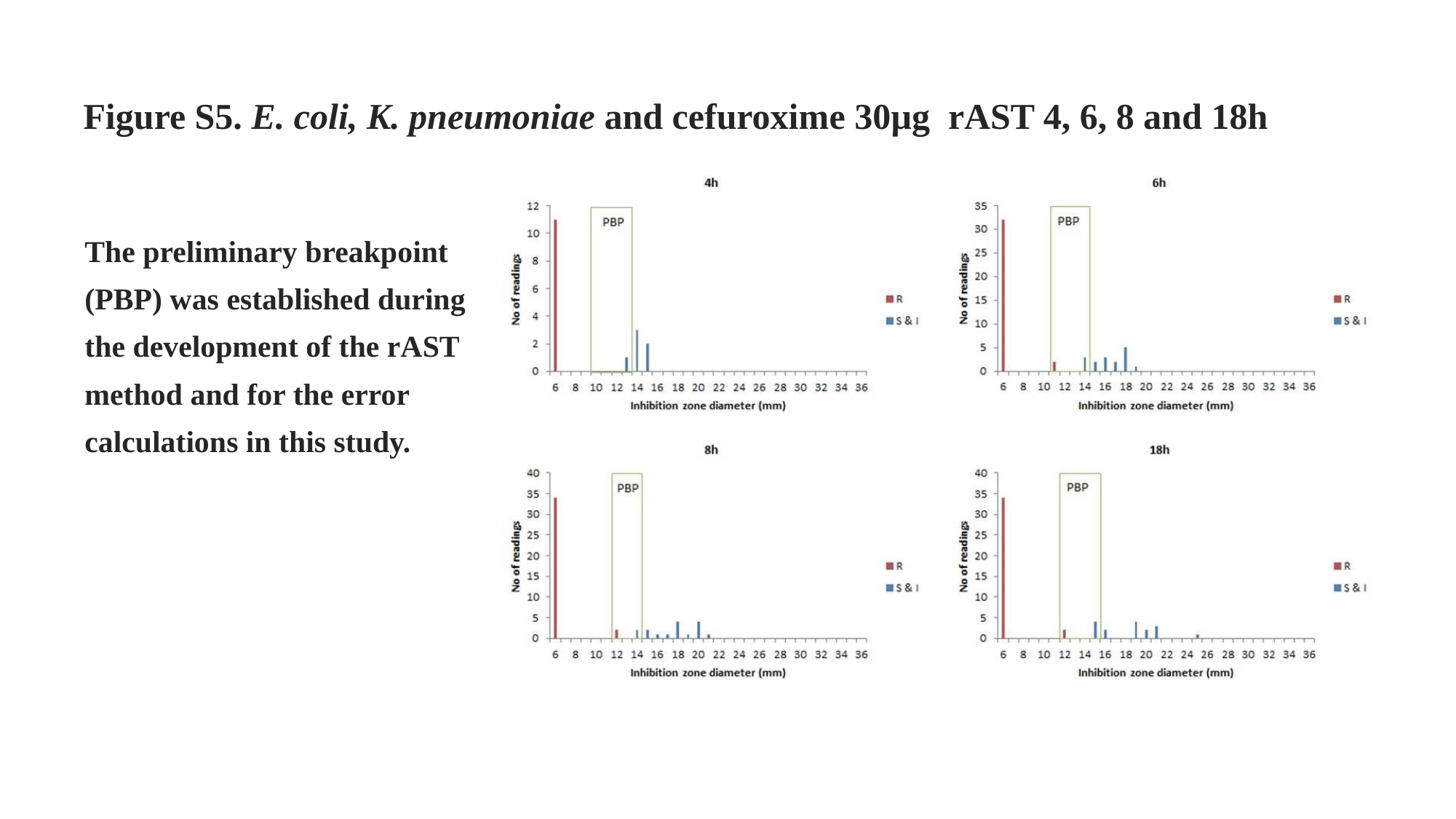

# Figure S5. E. coli, K. pneumoniae and cefuroxime 30µg rAST 4, 6, 8 and 18h
The preliminary breakpoint (PBP) was established during the development of the rAST method and for the error calculations in this study.

## Slide 10
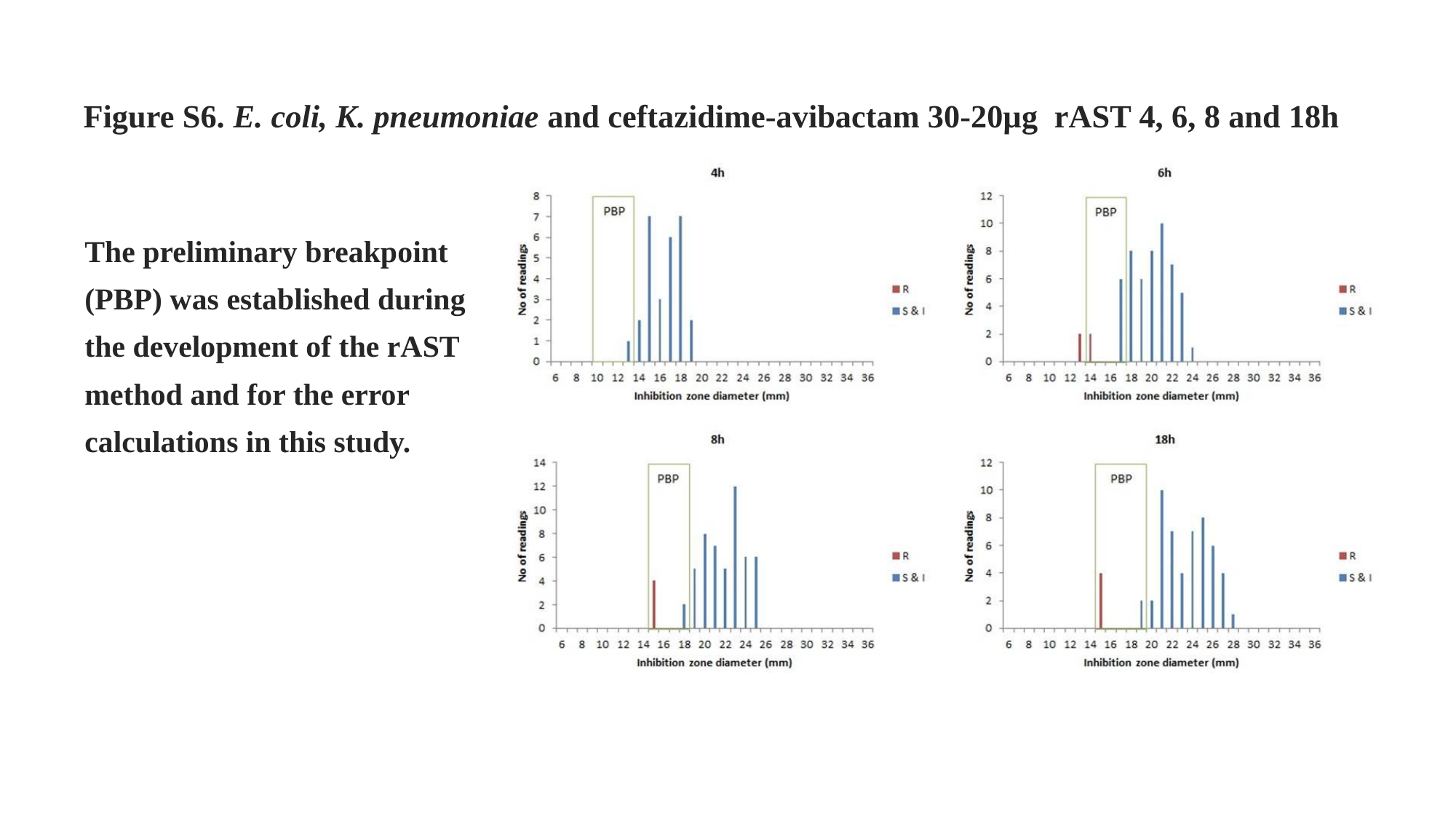

# Figure S6. E. coli, K. pneumoniae and ceftazidime-avibactam 30-20µg rAST 4, 6, 8 and 18h
The preliminary breakpoint (PBP) was established during the development of the rAST method and for the error calculations in this study.

## Slide 11
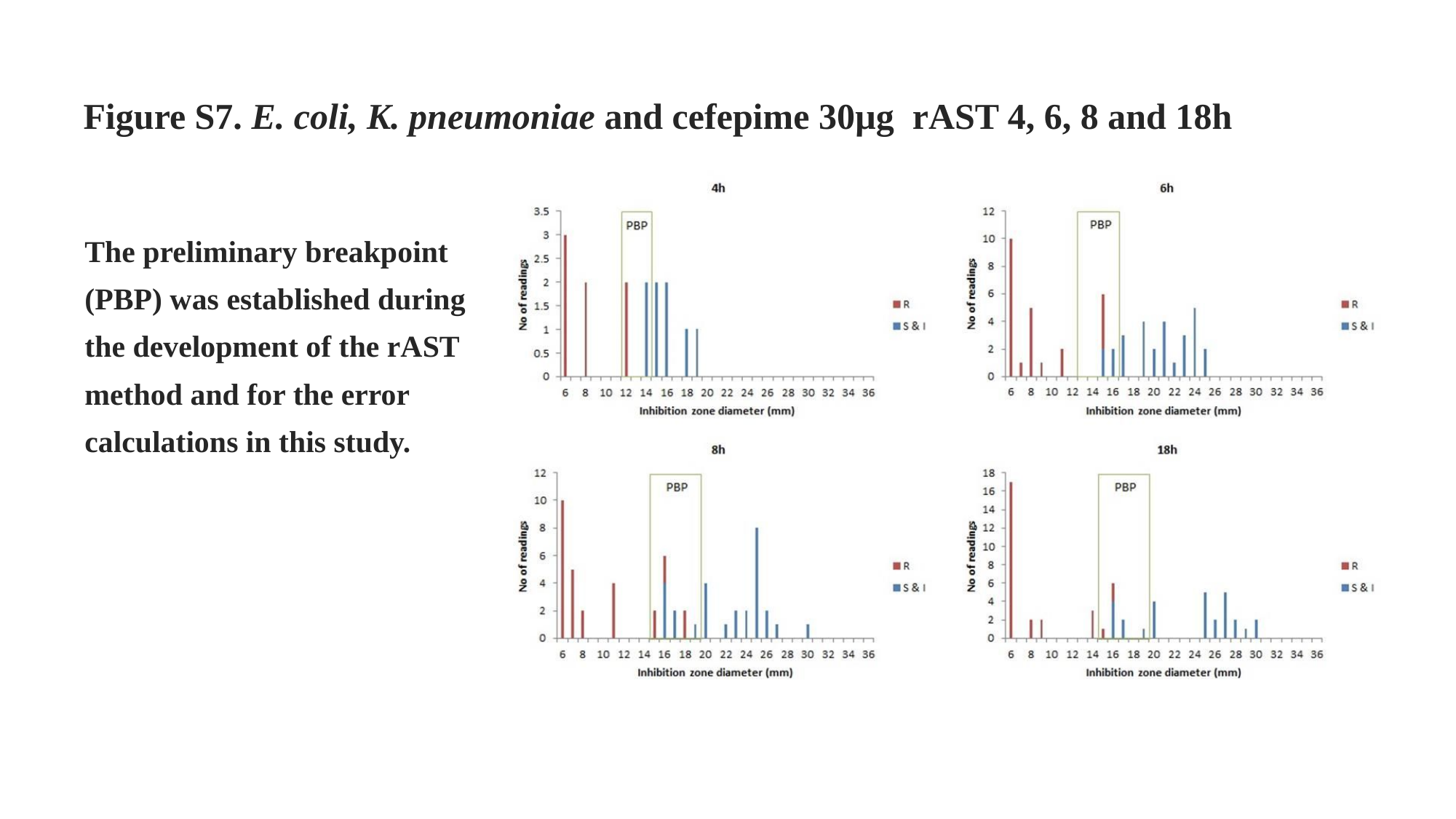

# Figure S7. E. coli, K. pneumoniae and cefepime 30µg rAST 4, 6, 8 and 18h
The preliminary breakpoint (PBP) was established during the development of the rAST method and for the error calculations in this study.

## Slide 12
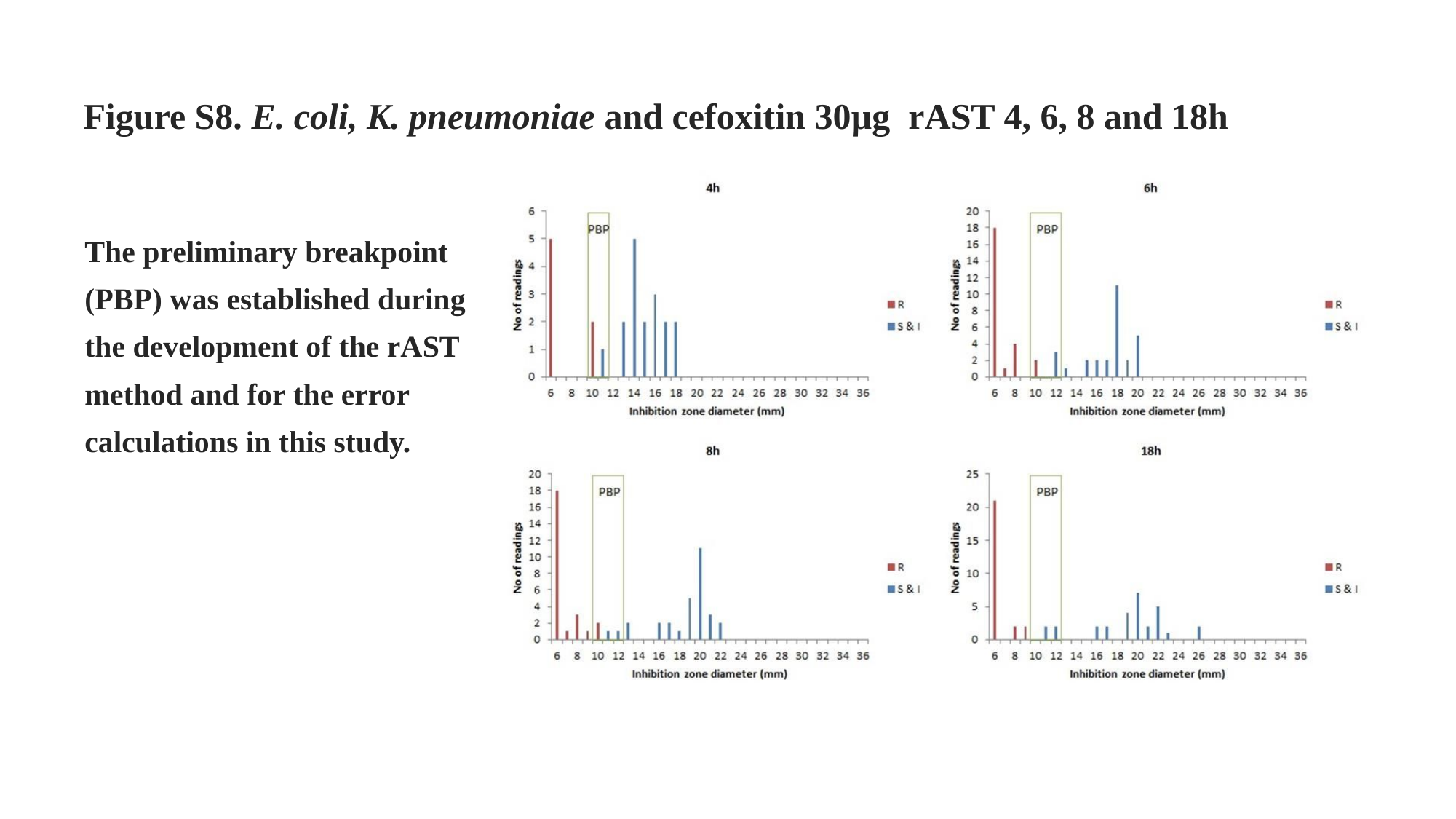

# Figure S8. E. coli, K. pneumoniae and cefoxitin 30µg rAST 4, 6, 8 and 18h
The preliminary breakpoint (PBP) was established during the development of the rAST method and for the error calculations in this study.

## Slide 13
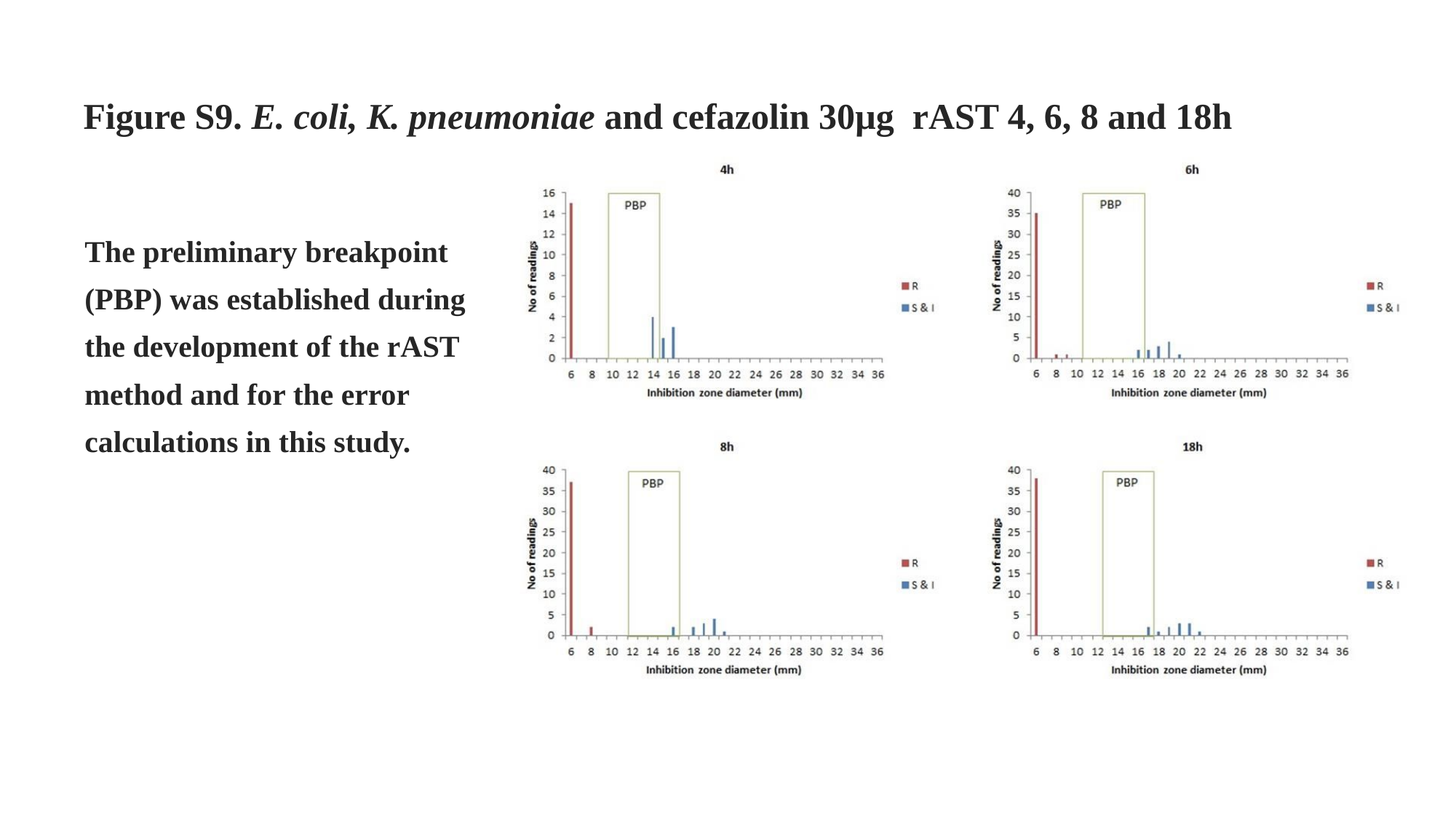

# Figure S9. E. coli, K. pneumoniae and cefazolin 30µg rAST 4, 6, 8 and 18h
The preliminary breakpoint (PBP) was established during the development of the rAST method and for the error calculations in this study.

## Slide 14
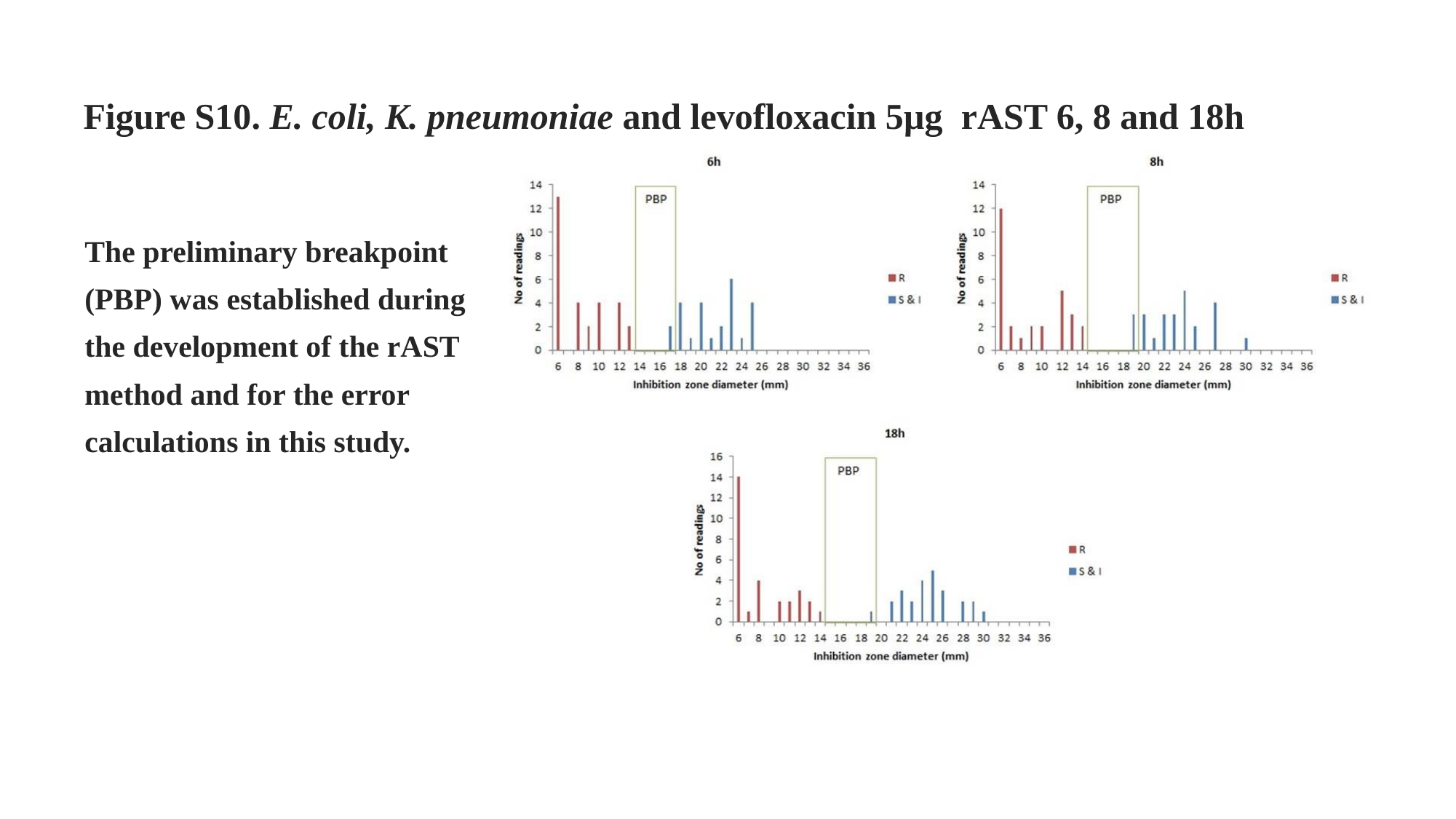

# Figure S10. E. coli, K. pneumoniae and levofloxacin 5µg rAST 6, 8 and 18h
The preliminary breakpoint (PBP) was established during the development of the rAST method and for the error calculations in this study.

## Slide 15
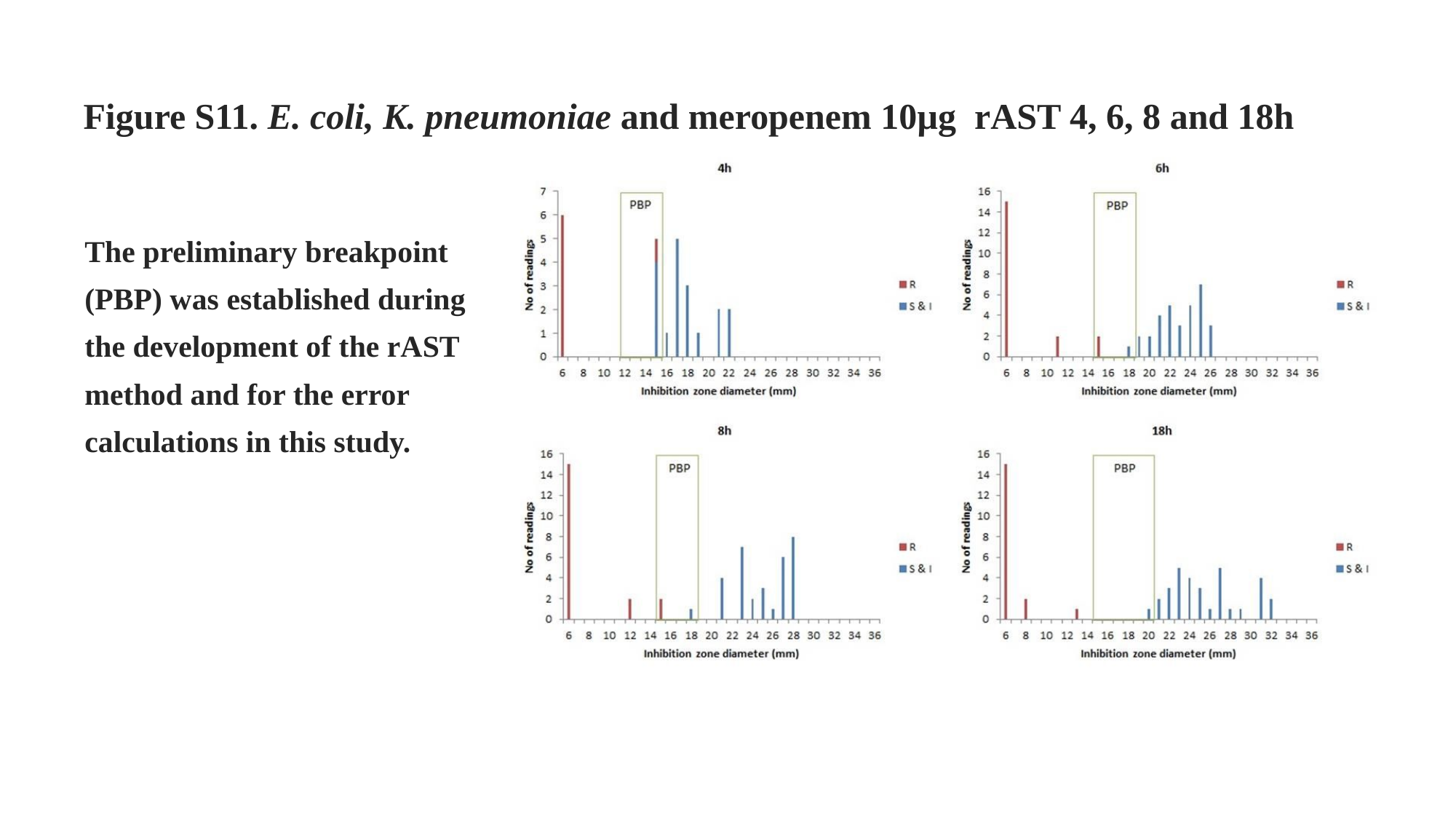

# Figure S11. E. coli, K. pneumoniae and meropenem 10µg rAST 4, 6, 8 and 18h
The preliminary breakpoint (PBP) was established during the development of the rAST method and for the error calculations in this study.

## Slide 16
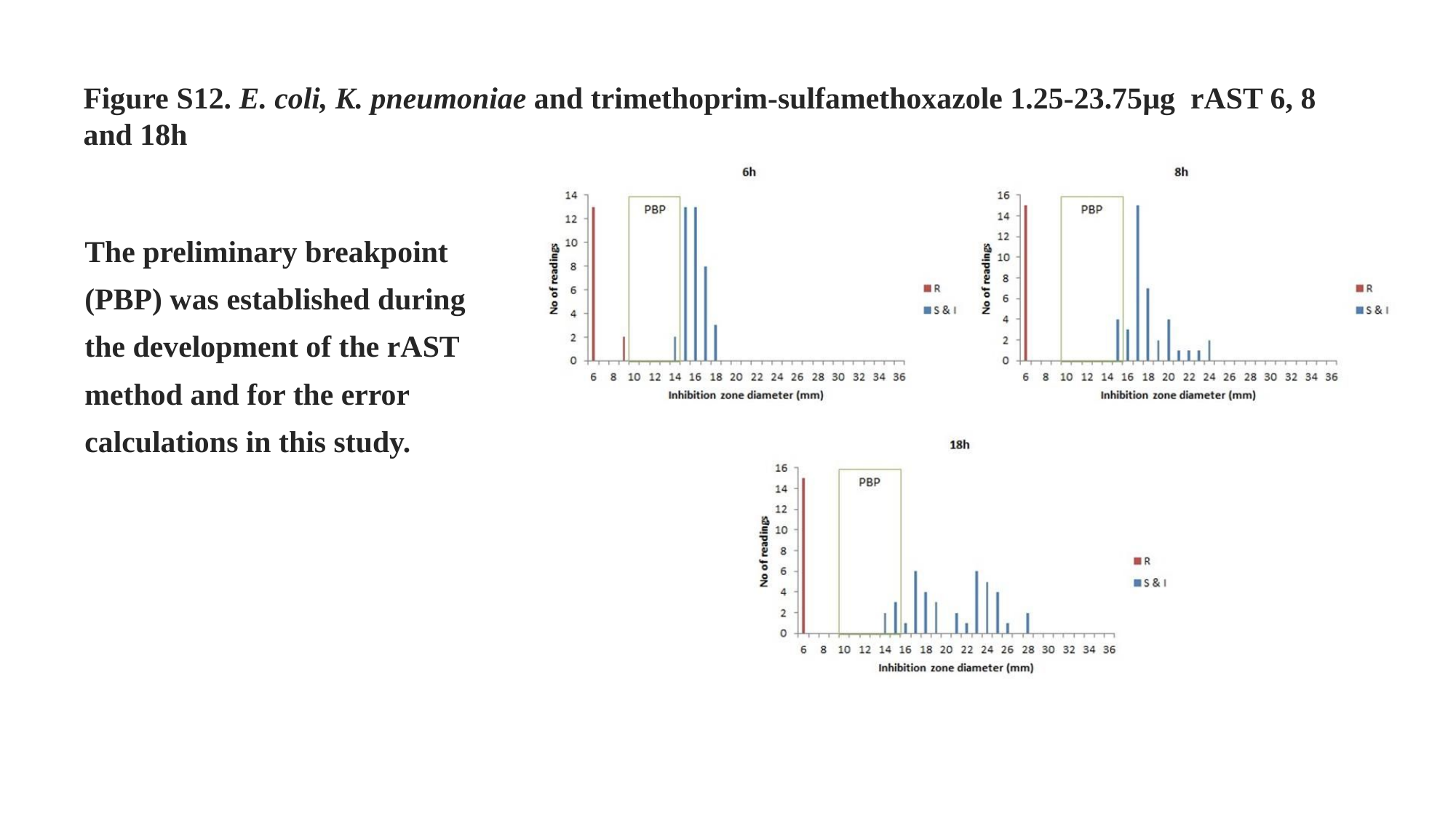

# Figure S12. E. coli, K. pneumoniae and trimethoprim-sulfamethoxazole 1.25-23.75µg rAST 6, 8 and 18h
The preliminary breakpoint (PBP) was established during the development of the rAST method and for the error calculations in this study.

## Slide 17
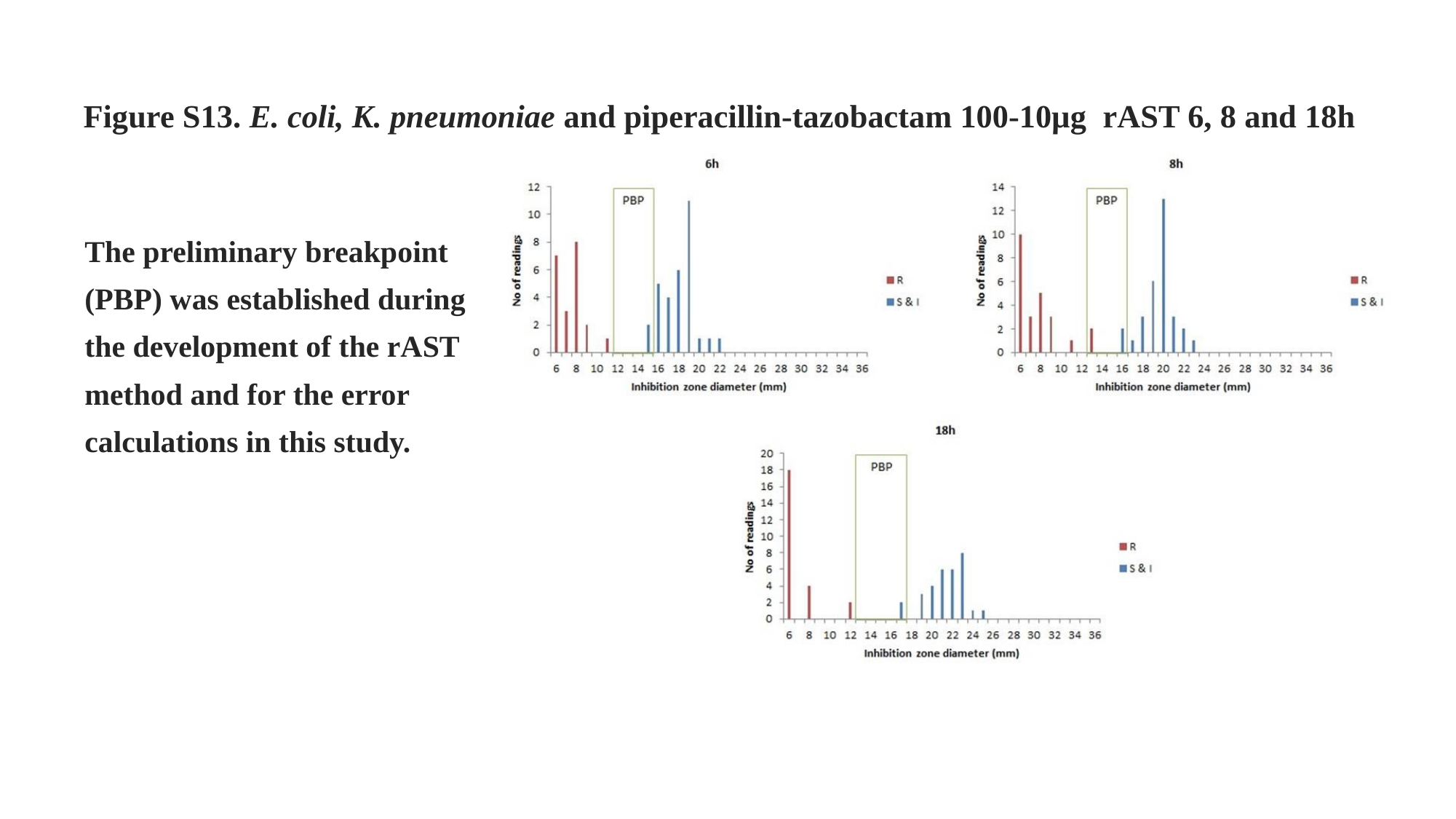

# Figure S13. E. coli, K. pneumoniae and piperacillin-tazobactam 100-10µg rAST 6, 8 and 18h
The preliminary breakpoint (PBP) was established during the development of the rAST method and for the error calculations in this study.

## Slide 18
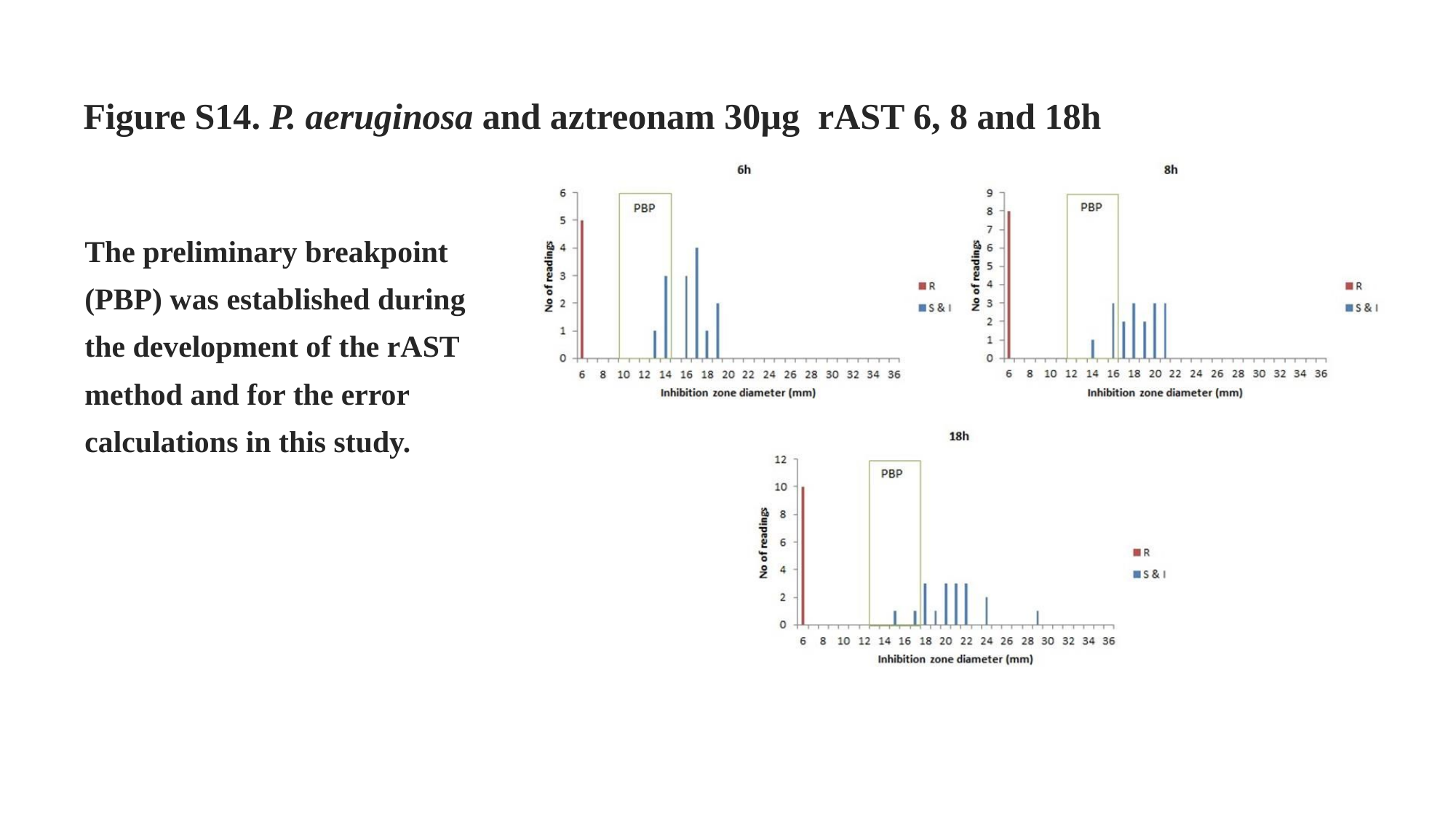

# Figure S14. P. aeruginosa and aztreonam 30µg rAST 6, 8 and 18h
The preliminary breakpoint (PBP) was established during the development of the rAST method and for the error calculations in this study.

## Slide 19
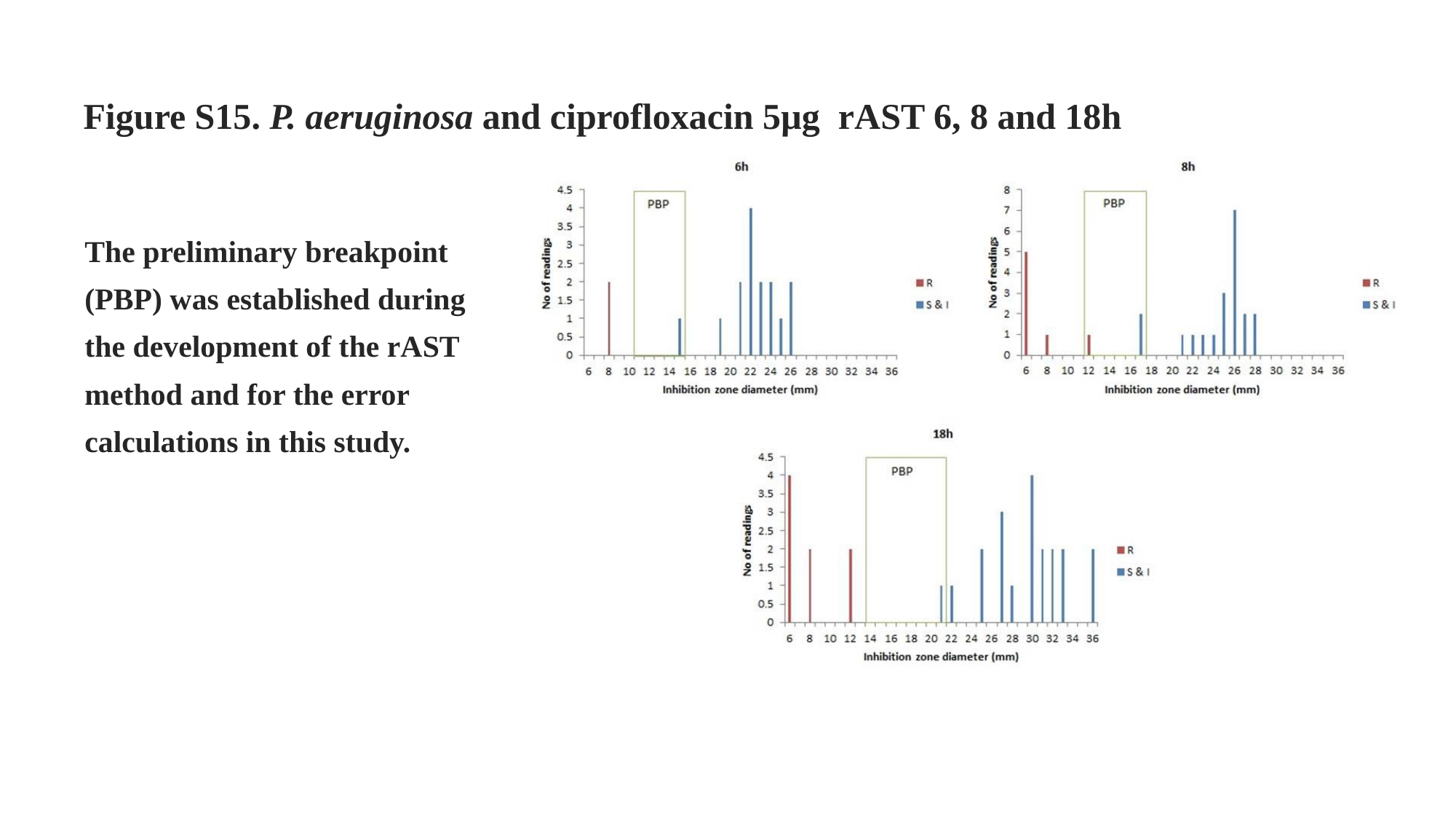

# Figure S15. P. aeruginosa and ciprofloxacin 5µg rAST 6, 8 and 18h
The preliminary breakpoint (PBP) was established during the development of the rAST method and for the error calculations in this study.

## Slide 20
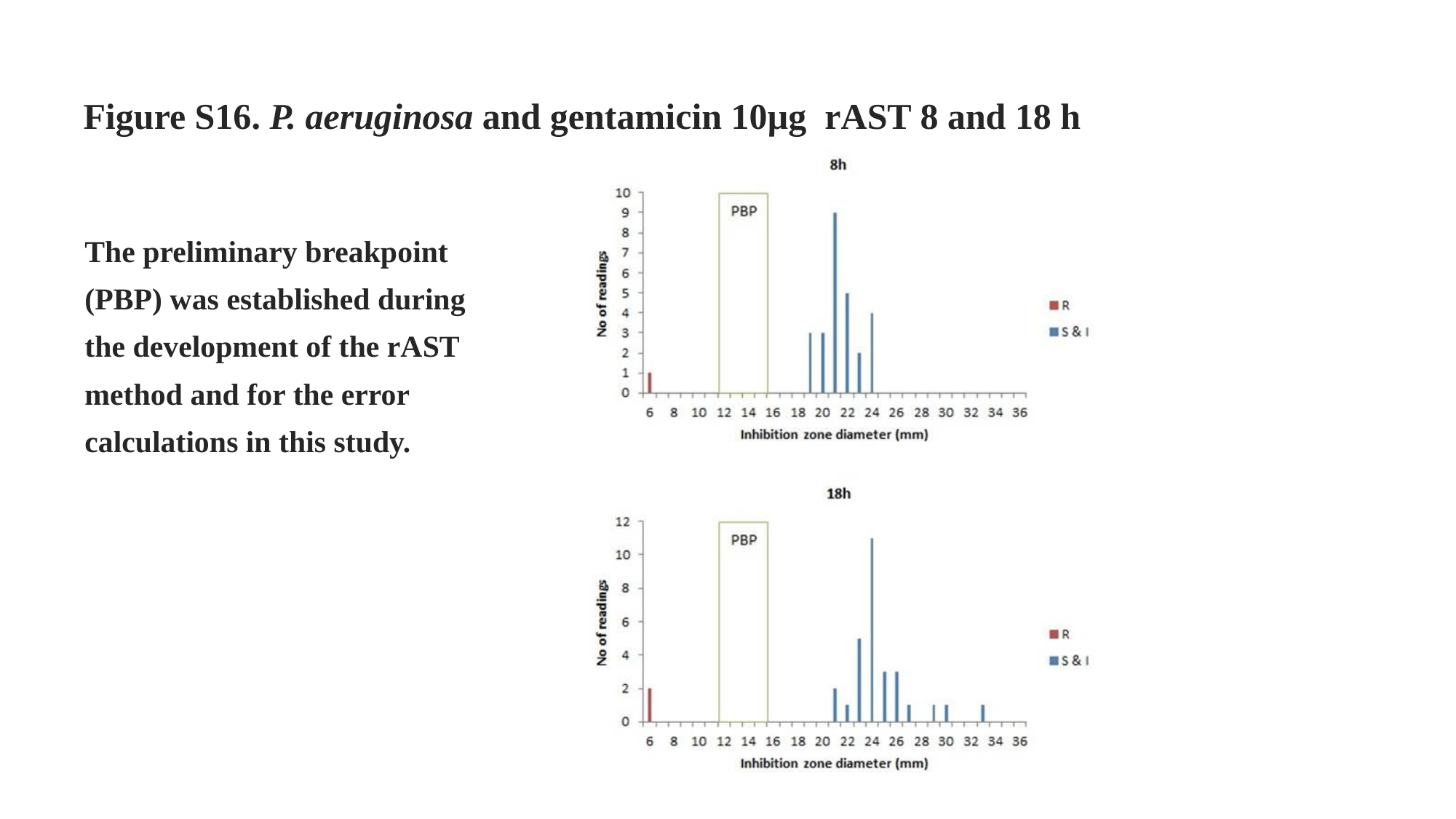

# Figure S16. P. aeruginosa and gentamicin 10µg rAST 8 and 18 h
The preliminary breakpoint (PBP) was established during the development of the rAST method and for the error calculations in this study.

## Slide 21
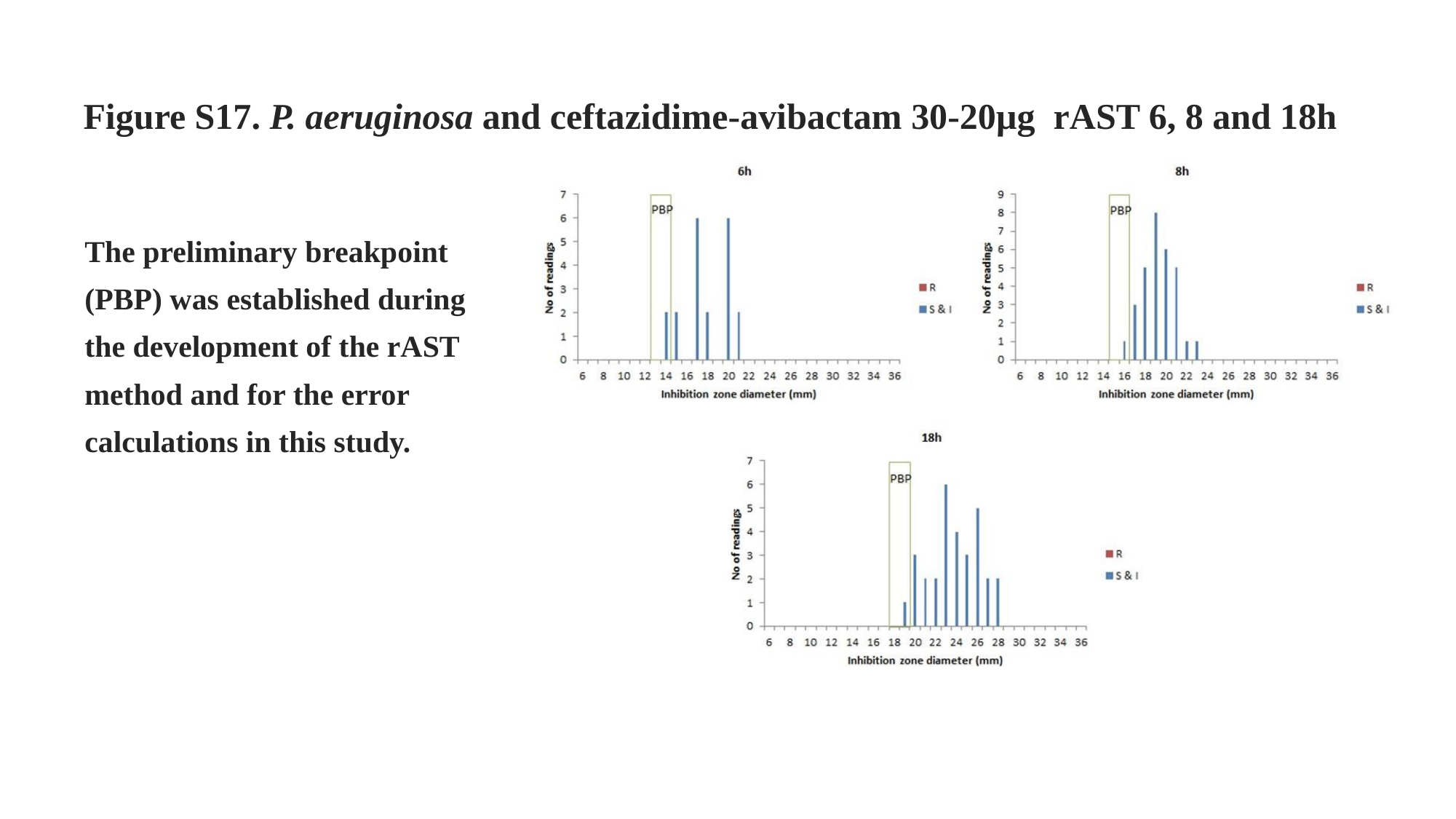

# Figure S17. P. aeruginosa and ceftazidime-avibactam 30-20µg rAST 6, 8 and 18h
The preliminary breakpoint (PBP) was established during the development of the rAST method and for the error calculations in this study.

## Slide 22
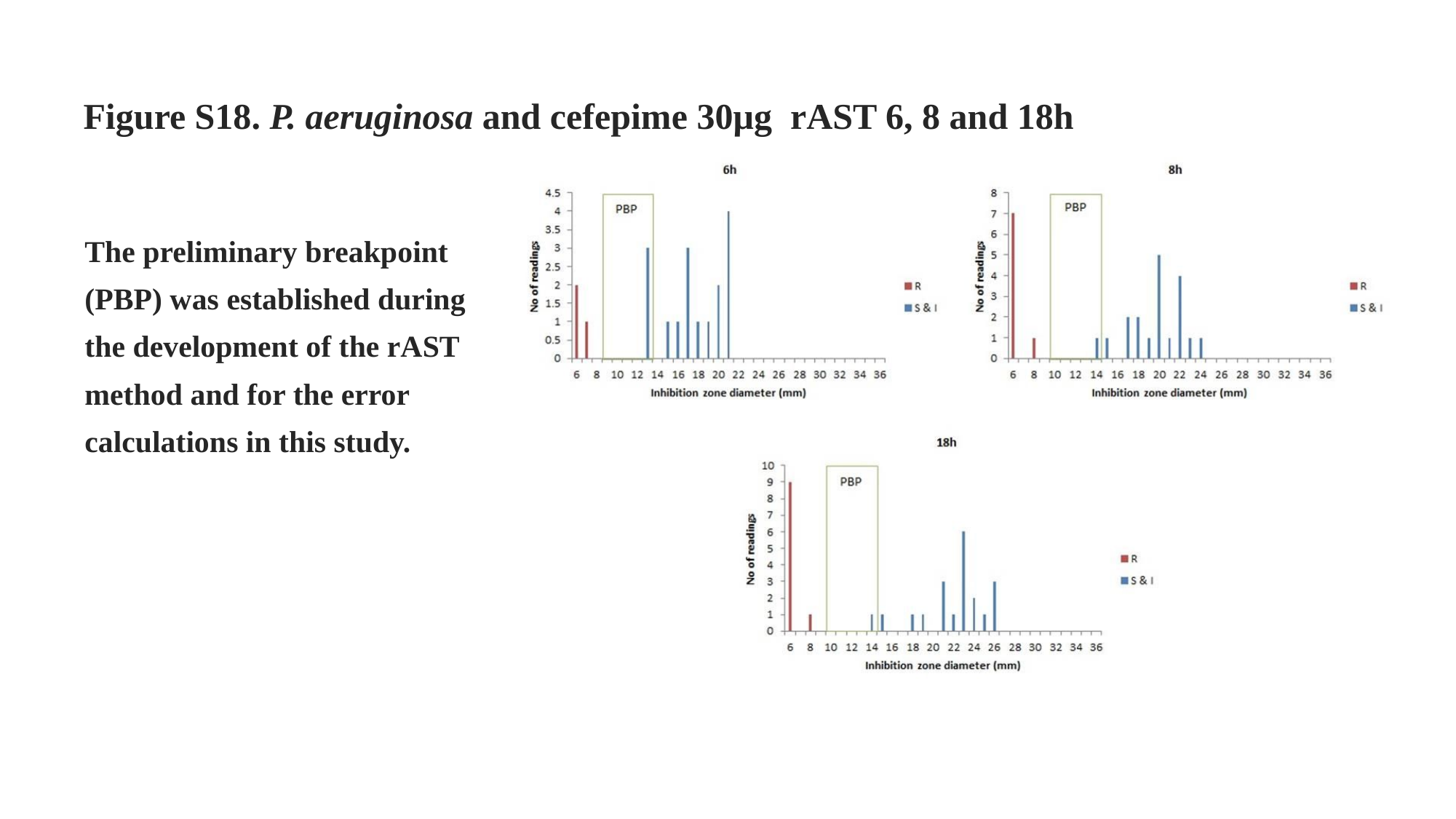

# Figure S18. P. aeruginosa and cefepime 30µg rAST 6, 8 and 18h
The preliminary breakpoint (PBP) was established during the development of the rAST method and for the error calculations in this study.

## Slide 23
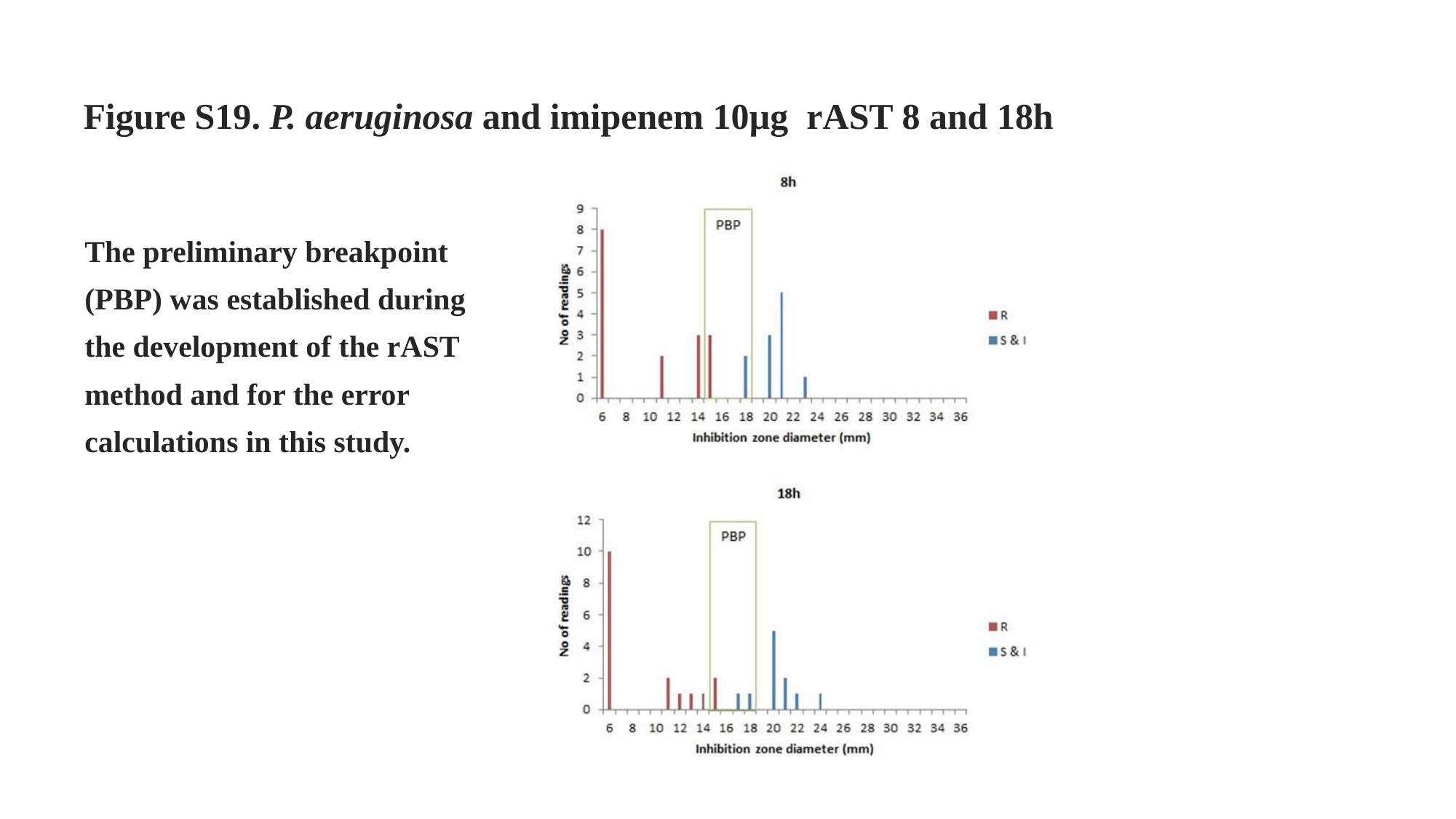

# Figure S19. P. aeruginosa and imipenem 10µg rAST 8 and 18h
The preliminary breakpoint (PBP) was established during the development of the rAST method and for the error calculations in this study.

## Slide 24
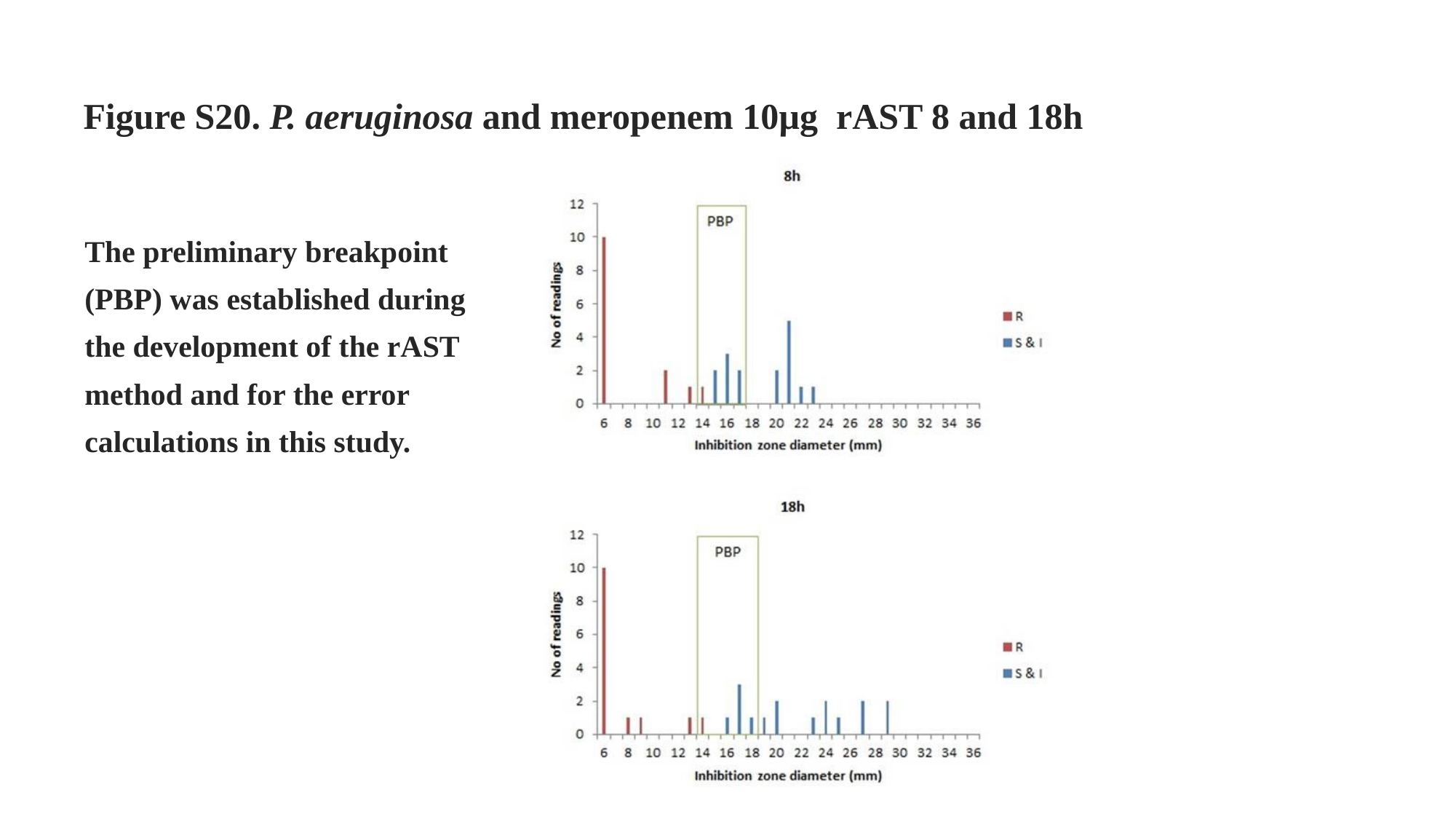

# Figure S20. P. aeruginosa and meropenem 10µg rAST 8 and 18h
The preliminary breakpoint (PBP) was established during the development of the rAST method and for the error calculations in this study.

## Slide 25
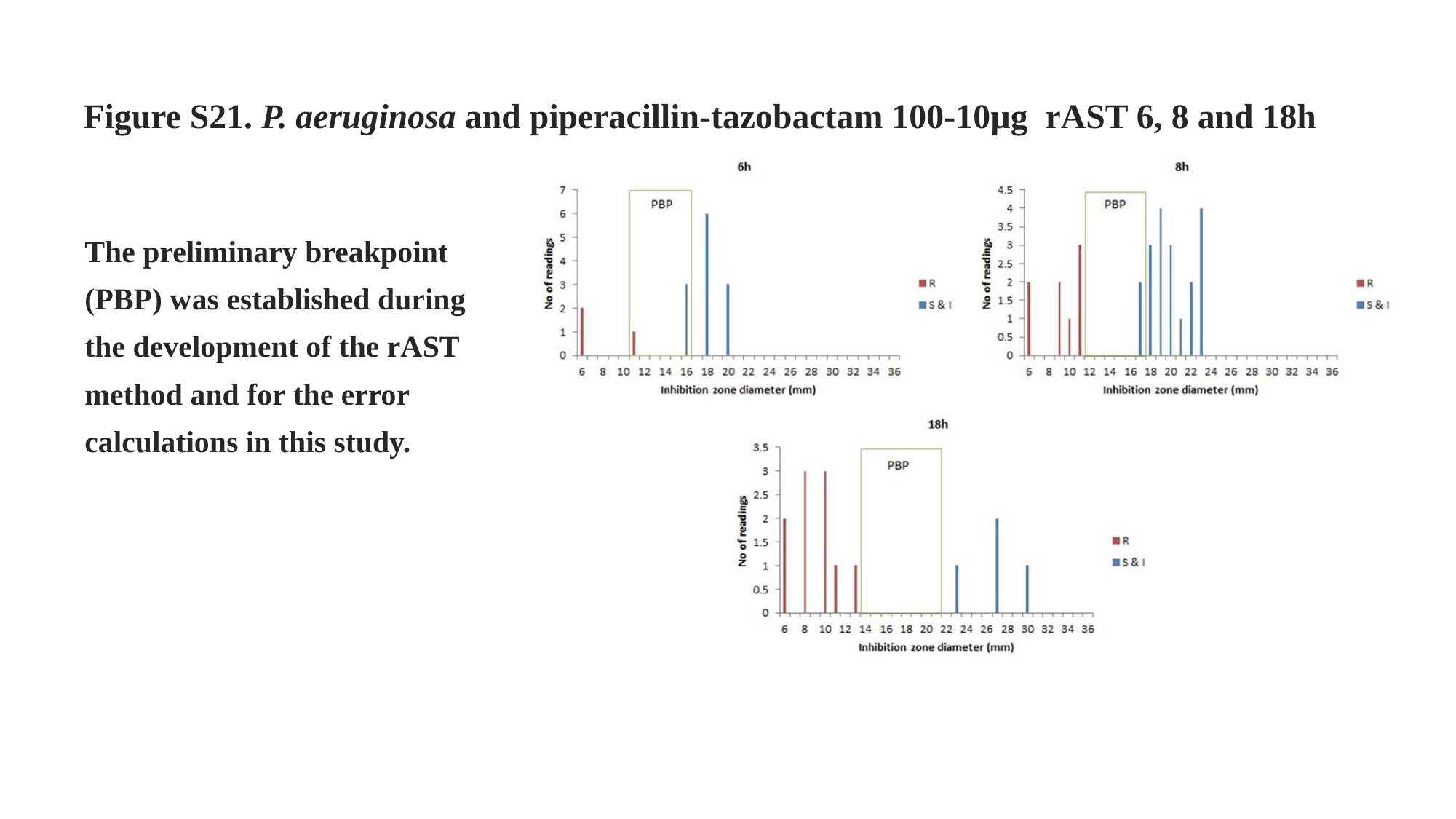

# Figure S21. P. aeruginosa and piperacillin-tazobactam 100-10µg rAST 6, 8 and 18h
The preliminary breakpoint (PBP) was established during the development of the rAST method and for the error calculations in this study.

## Slide 26
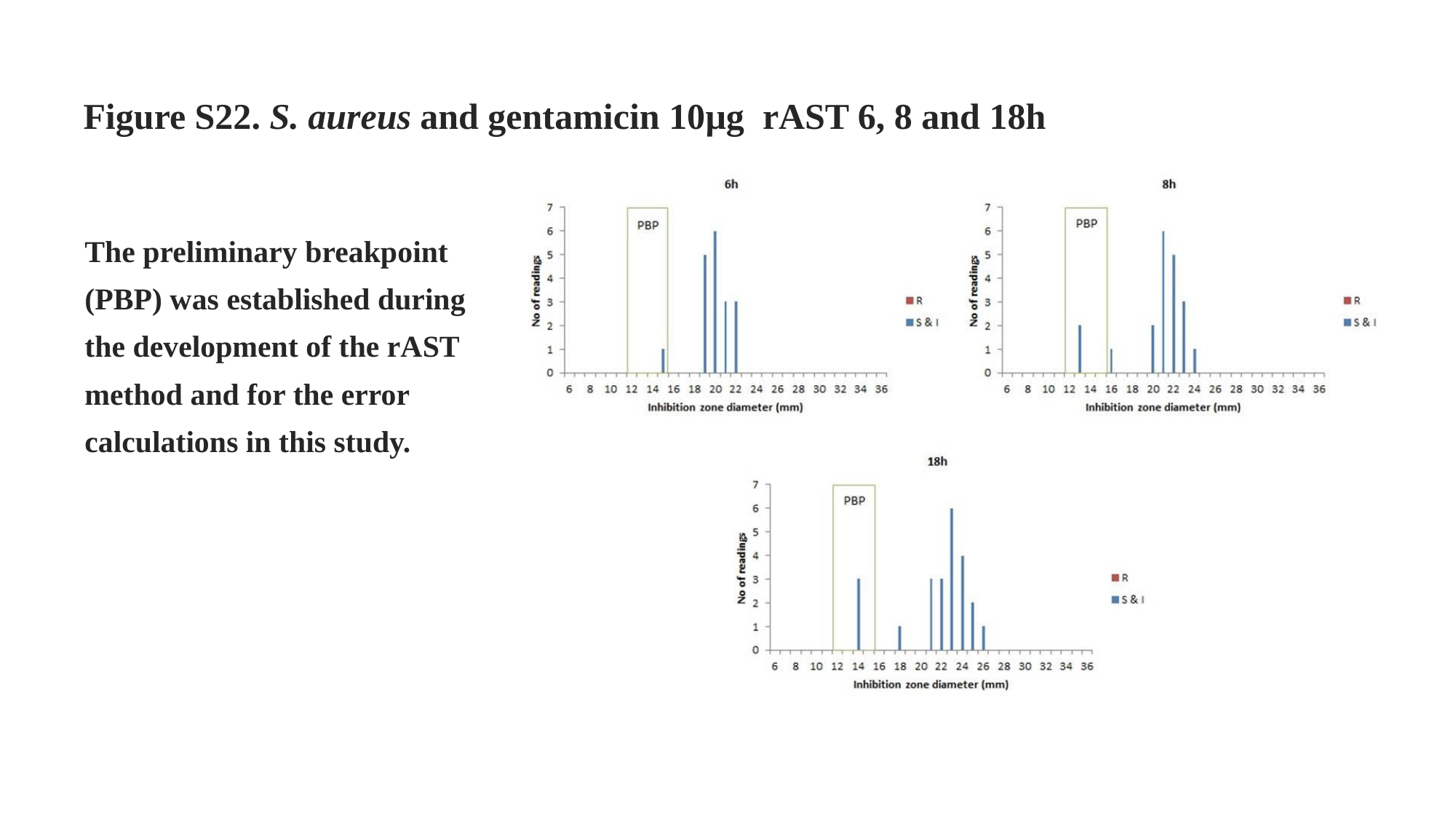

# Figure S22. S. aureus and gentamicin 10µg rAST 6, 8 and 18h
The preliminary breakpoint (PBP) was established during the development of the rAST method and for the error calculations in this study.

## Slide 27
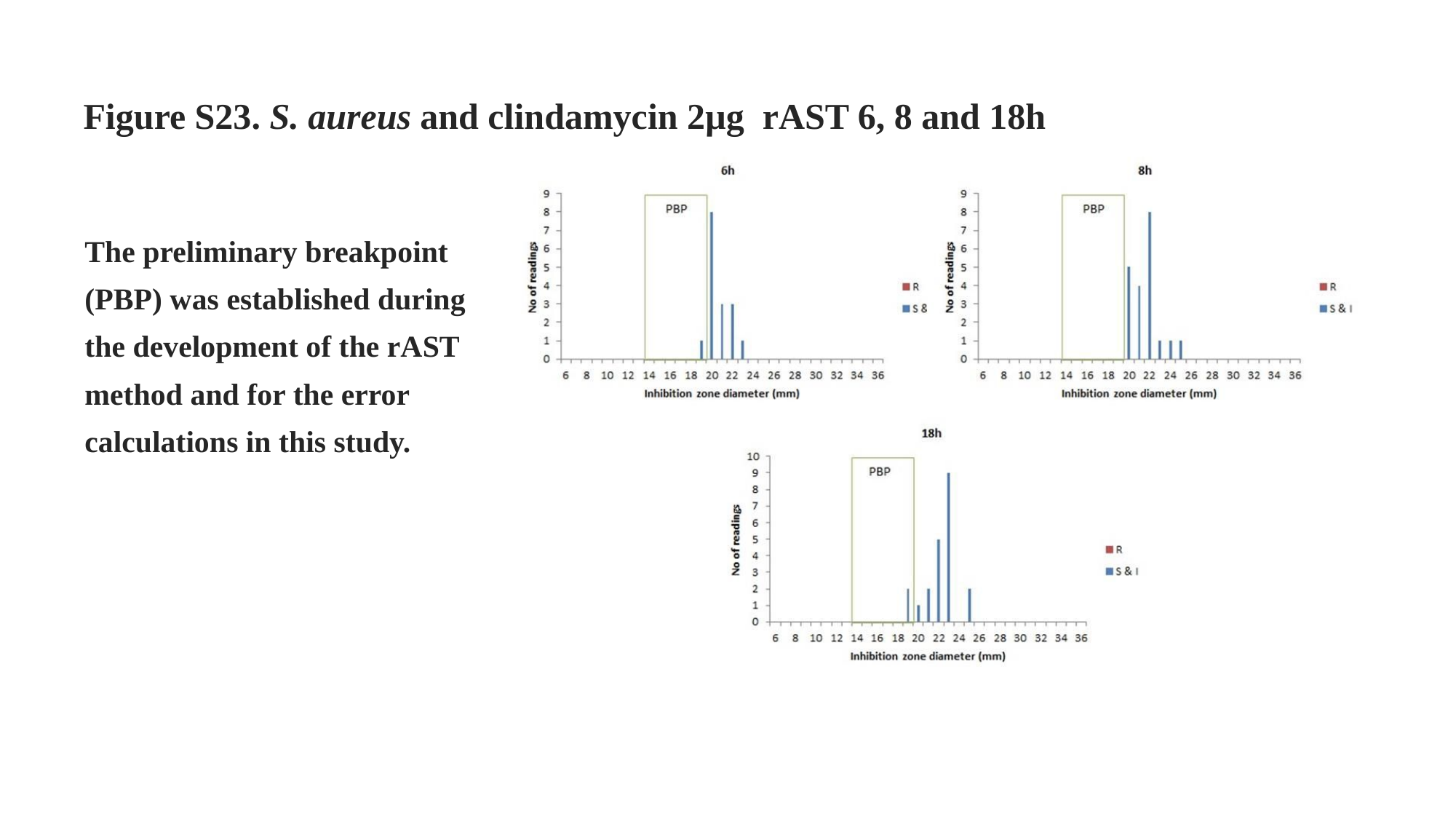

# Figure S23. S. aureus and clindamycin 2µg rAST 6, 8 and 18h
The preliminary breakpoint (PBP) was established during the development of the rAST method and for the error calculations in this study.

## Slide 28
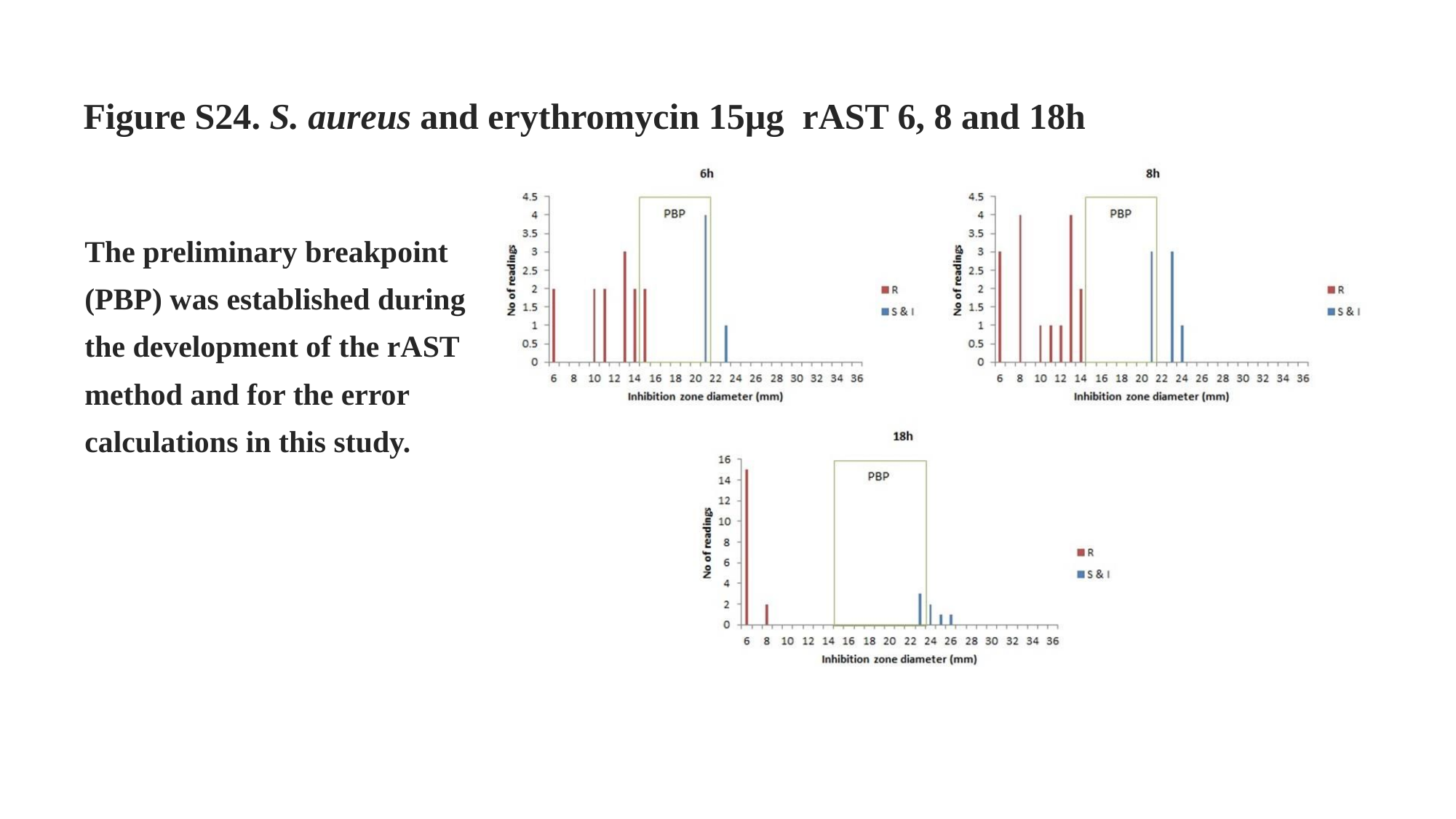

# Figure S24. S. aureus and erythromycin 15µg rAST 6, 8 and 18h
The preliminary breakpoint (PBP) was established during the development of the rAST method and for the error calculations in this study.

## Slide 29
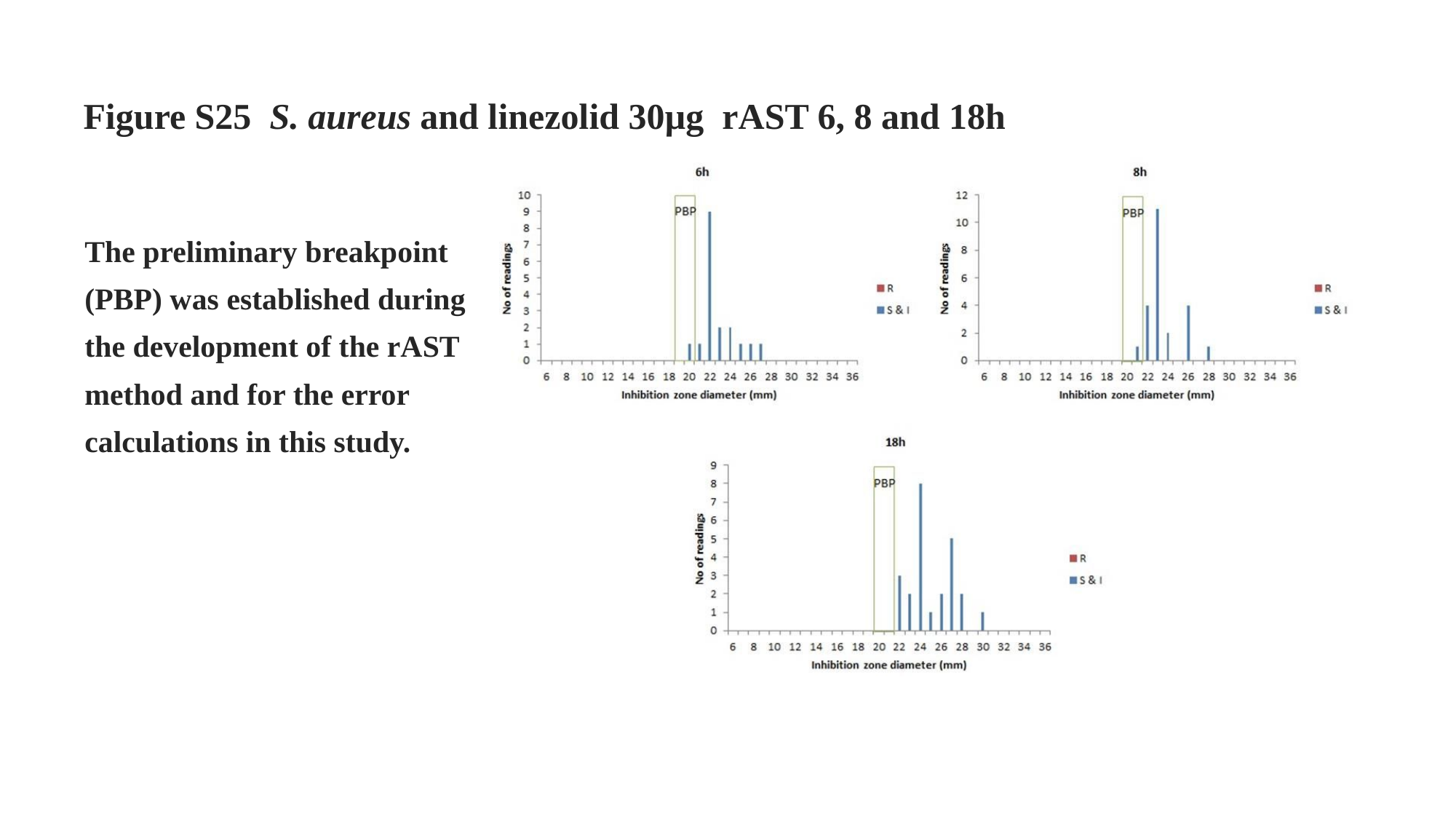

# Figure S25 S. aureus and linezolid 30µg rAST 6, 8 and 18h
The preliminary breakpoint (PBP) was established during the development of the rAST method and for the error calculations in this study.

## Slide 30
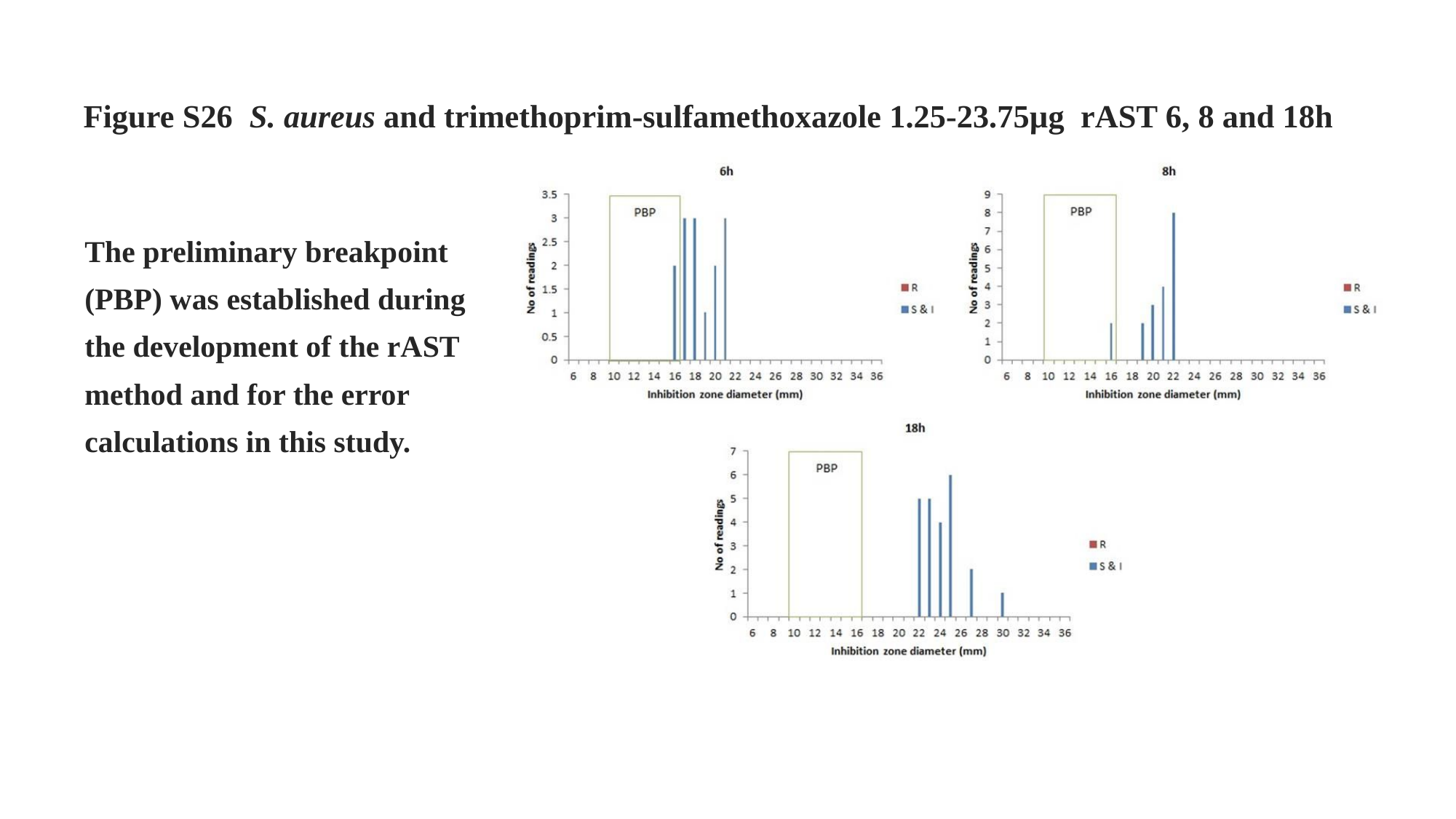

# Figure S26 S. aureus and trimethoprim-sulfamethoxazole 1.25-23.75µg rAST 6, 8 and 18h
The preliminary breakpoint (PBP) was established during the development of the rAST method and for the error calculations in this study.

## Slide 31
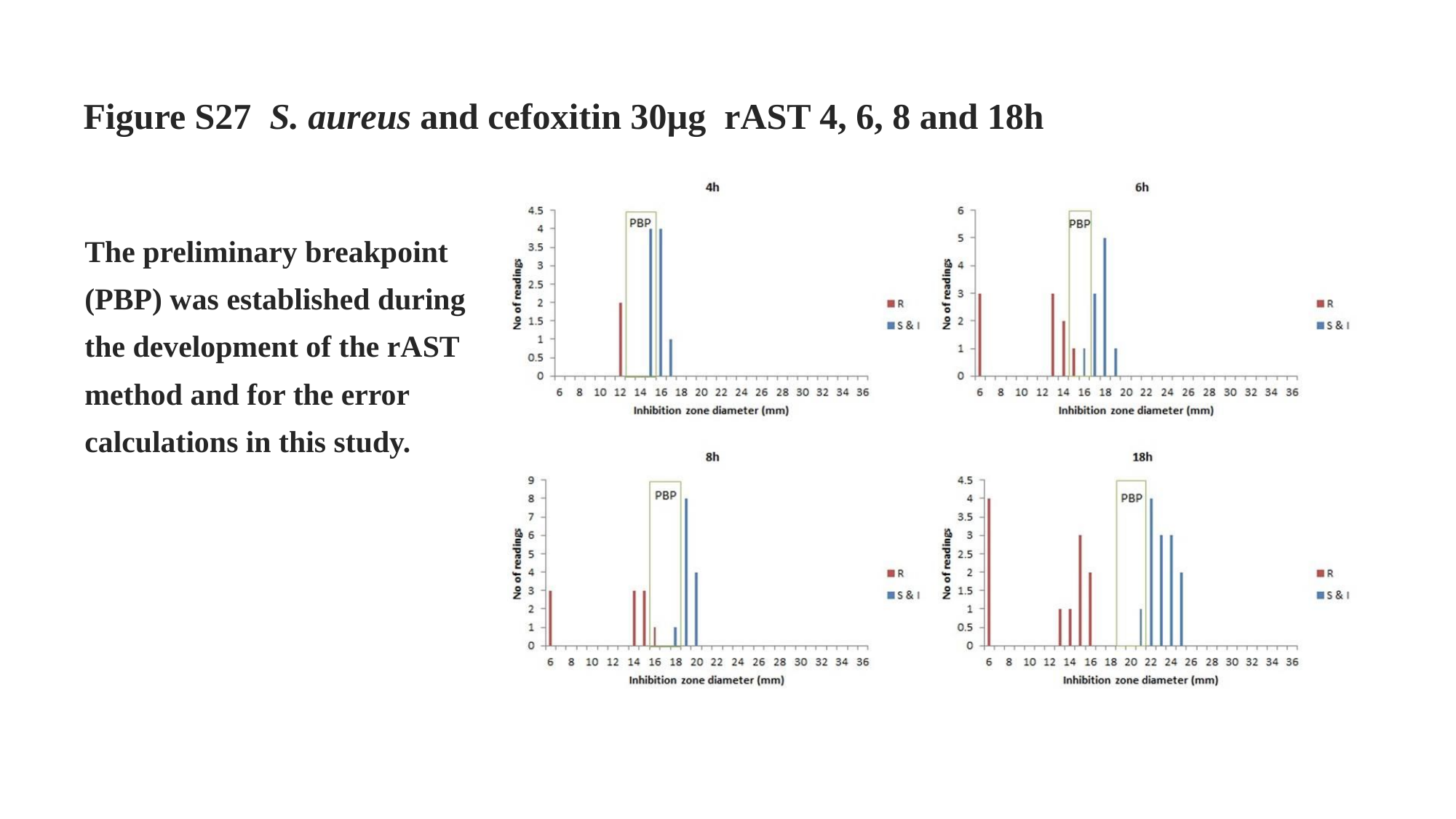

# Figure S27 S. aureus and cefoxitin 30µg rAST 4, 6, 8 and 18h
The preliminary breakpoint (PBP) was established during the development of the rAST method and for the error calculations in this study.

## Slide 32
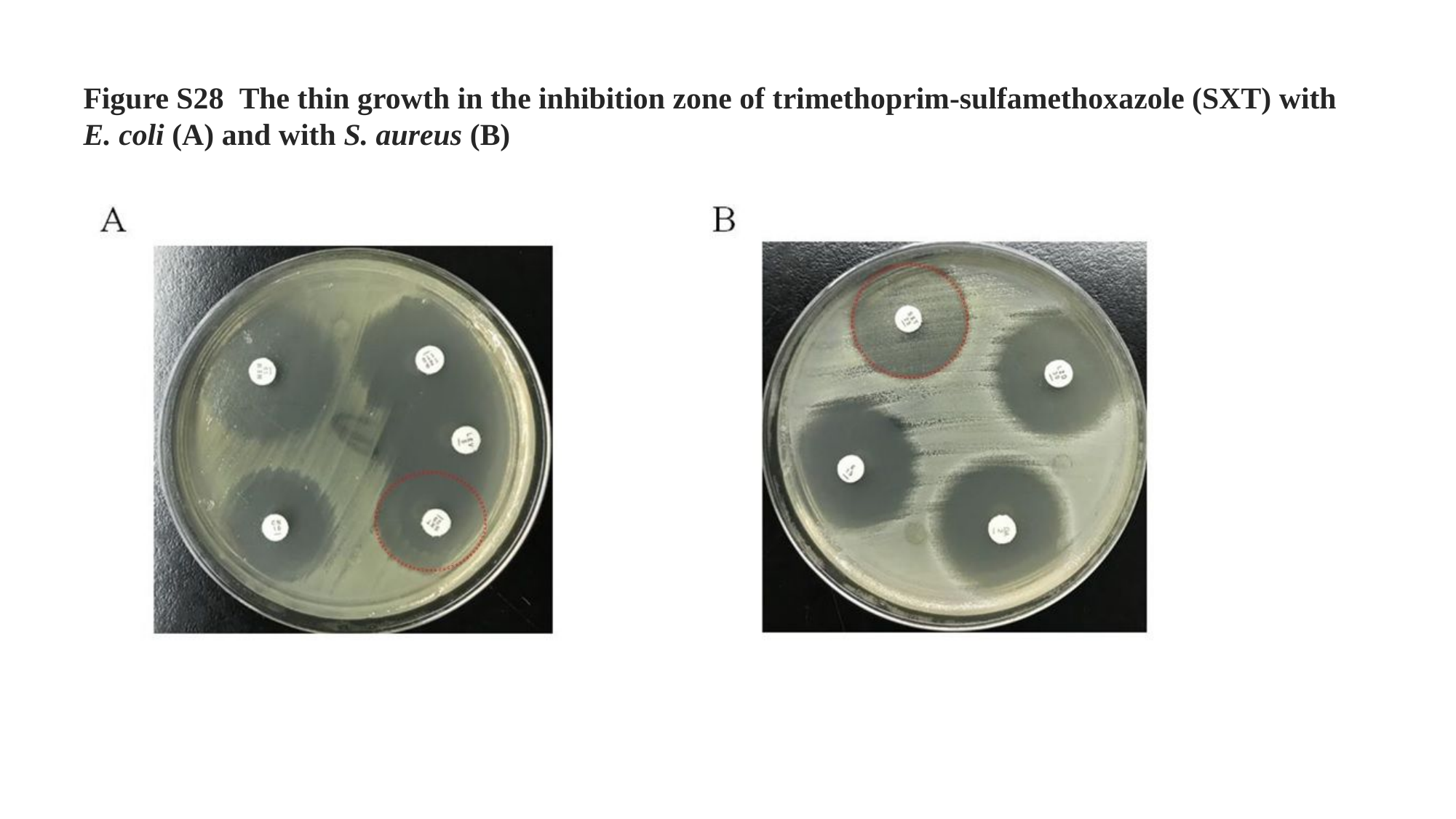

# Figure S28 The thin growth in the inhibition zone of trimethoprim-sulfamethoxazole (SXT) with E. coli (A) and with S. aureus (B)
